# Psychometric properties of the EQ-5D-5L: a systematic review of the literature

**DOI:** 10.1007/s11136-020-02688-y

**Published:** 2020-12-07

**Authors:** You-Shan Feng, Thomas Kohlmann, Mathieu F. Janssen, Ines Buchholz

**Affiliations:** 1 Institute for Community Medicine, Medical University Greifswald, Greifswald, Germany; 2 Institute for Clinical Epidemiology and Applied Biometrics, Medical University of Tübingen, Silcherstraße 5, 72076 Tübingen, Germany; 3 Section Medical Psychology and Psychotherapy, Department of Psychiatry, Erasmus MC, Erasmus University, Rotterdam, The Netherlands

**Keywords:** EQ-5D, EQ-5D-5L, Systematic review, Health-Related Quality of Life, Psychometric properties

## Abstract

**Purpose:**

Although the EQ-5D has a long history of use in a wide range of populations, the newer five-level version (EQ-5D-5L) has not yet had such extensive experience. This systematic review summarizes the available published scientific evidence on the psychometric properties of the EQ-5D-5L.

**Methods:**

Pre-determined key words and exclusion criteria were used to systematically search publications from 2011 to 2019. Information on study characteristics and psychometric properties were extracted: specifically, EQ-5D-5L distribution (including ceiling and floor), missing values, reliability (test–retest), validity (convergent, known-groups, discriminate) and responsiveness (distribution, anchor-based). EQ-5D-5L index value means, ceiling and correlation coefficients (convergent validity) were pooled across the studies using random-effects models.

**Results:**

Of the 889 identified publications, 99 were included for review, representing 32 countries. Musculoskeletal/orthopedic problems and cancer (*n* = 8 each) were most often studied. Most papers found missing values (17 of 17 papers) and floor effects (43 of 48 papers) to be unproblematic. While the index was found to be reliable (9 of 9 papers), individual dimensions exhibited instability over time. Index values and dimensions demonstrated moderate to strong correlations with global health measures, other multi-attribute utility instruments, physical/functional health, pain, activities of daily living, and clinical/biological measures. The instrument was not correlated with life satisfaction and cognition/communication measures. Responsiveness was addressed by 15 studies, finding moderate effect sizes when confined to studied subgroups with improvements in health.

**Conclusions:**

The EQ-5D-5L exhibits excellent psychometric properties across a broad range of populations, conditions and settings. Rigorous exploration of its responsiveness is needed.

**Electronic supplementary material:**

The online version of this article (10.1007/s11136-020-02688-y) contains supplementary material, which is available to authorized users.

## Background

The EQ-5D is a broadly used generic multi-attribute health utility instrument. In addition to a thermometer-like visual analog scale (VAS) anchored by 0 (worst imaginable health) and 100 (best imaginable health), the EQ-5D’s descriptive system comprises five dimensions with one item per dimension: mobility (MO), self-care (SC), usual activities (UA), pain/discomfort (PD) and anxiety/depression (AD). Responses to these items can be converted into a single measure of health utility using preference-based (typically country-specific) weights. Preference weights are derived from preference elicitation studies using hypothetical EQ-5D health profiles [1], typically sampling a general population.

Until 2005, respondents could select from three response levels of function or symptoms for each dimension (the EQ-5D-3L; 3L). However, due to evidence of notable ceiling effects of the EQ-5D-3L in some populations [2–5] and concerns regarding the instrument’s sensitivity to certain patient-relevant changes [6–10], a five response level version of the instrument was developed by the EuroQol group in 2010 [11, 12]. The five-level version (EQ-5D-5L; 5L) added two response levels: one between “no problems” (level 1) and “moderate/some problems” (level 2 in 3L, level 3 in 5L), and another one between “moderate/some problems” and “severe problems” (level 3 in 3L, level 5 in 5L). The EQ-5D-5L also updated the middle response level with the term “moderate” from the EQ-5D-3L’s “some” for the first three dimensions, while the most severe response level for MO was changed from “confined to bed” to “unable to walk about”. Additionally, the instructions for marking overall health today on the visual analog scale (VAS) were different between the two versions until 2019. The EQ-5D-5L is currently available for more than 130 languages [13] and has been formally tested against the EQ-5D-3L in numerous studies, demonstrating improved psychometric properties over the EQ-5D-3L [14]. An interim scoring strategy that applies existing EQ-5D-3L preference weights to EQ-5D-5L can be used if EQ-5D-5L preference weights for certain populations are not yet available [A4].

Although its use has expanded to a wide range of settings and research purposes, there is no study reporting a comprehensive review of the measurement properties of the EQ-5D-5L. This review will be informative for researchers interested in economic evaluation and preference measurement, decision makers, users of EQ-5D-5L as patient-reported outcome measure for improving health care, and readers who need to interpret the findings from studies incorporating the EQ-5D-5L. The 5L instrument has now enjoyed over a decade of use and this paper aims to summarize the existing evidence on the psychometric properties of the EQ-5D-5L. A second objective of this review is to identify knowledge gaps regarding the psychometric properties of the EQ-5D-5L, and to highlight important areas for future research.

## Methods

This literature search and review was guided by the PRISMA guidance on systematic reviews and meta-analyses [15]. This review focuses on the descriptive system of the EQ-5D-5L (the five items) as it was not always clear which version of the EQ-VAS was used in extracted studies.

### Literature search

Four online databases—PUBMED (MEDLINE), PsycINFO, Excerpta Medica Database (EMBASE), and the EuroQol website—were searched using pre-determined terms: “EQ-5D,” “EQ-5D-5L,” “5L,” “EuroQol” and “5 Level.” The search included publications up to January 2019. Duplicates were assessed using author names, titles and journals. Exact search strategy and terms can be found in Supplementary Table 1.

Two screening phases were conducted: (1) title and abstract, and (2) full text. Two researchers experienced in psychometric research methods and the EQ-5D instruments (IB and YF) independently screened the publications and reached consensus on any disagreements to determine inclusion. When consensus could not be reached, two senior researchers with extensive experience in psychometric research, health-related quality of life (HRQoL) measurement and the EQ-5D instrument were consulted for a final decision (TK and MFJ).

The a priori exclusion criteria were:

1. does not study humans 18 years or older;
2. publication language is other than German or English;
3. study does not assess the official version of the EQ-5D-5L or an experimental version of the 5L was used;
4. published prior to 2005 (prior to development of the 5L);
5. not a peer-reviewed primary study, literature review or conference paper (conference papers were included but other conference proceedings such as presentations or posters were excluded); and
6. not evaluating the measurement and psychometric properties of the EQ-5D-5L.

### Data extraction

Publications selected for inclusion were reviewed and data entered into pre-determined tables by either YF or IB. Sometimes, values needed to be estimated from available information. When information on means and standard deviations were not available, but other sufficient data were reported (such as range or median), the mean and standard deviations were estimated using recommendations from Wan et al. 2014 [16]. When multiple studies use the same underlying dataset, data was extracted only once (e.g., [A20, A26, A31, A36–A38, A49, A53, A77, A79, A96]). General study characteristics including sample size, study design, sample characteristics and version of EQ-5D-5L were extracted, as were information on distributional properties such as means, percent reporting best health (“no problems” on dimensions or ‘11111’ across the health profile), percent reporting worst health (“extreme” or “unable to” on dimensions or ‘55555’ across the health profile) and missing values, for dimensions as well as the health profile. Although no guidance for level of missing values indicate the feasibility of an instrument, ≤ 5% has been found to be acceptable for multiple imputation [17]. Missing values ≤ 5% and floor ≤ 15% are considered acceptable [18].

*Reliability* is the consistency of an instrument, internally (extent to which subscale items are interrelated) as well as the instrument’s stability across time (whether the instrument produces similar results in stable environments). Internal consistency is not a relevant psychometric property for the EQ-5D instruments and therefore we did not include it in this review. Agreement between two applications of the instrument over a period of time over which it should be stable (test–retest) is usually evaluated using Cohen’s Kappa (*κ*) for categorical items (EQ-5D-5L items) or ICC for continuous values (EQ-5D-5L index value), with a level of ≥0.8 and ≥0.7 determined as acceptable, respectively [19–21]. We relied on the guidance from Cicchetti 1994 [22] to define Kappa and ICC: < 0.40 = poor, 0.40–0.59 = fair, 0.60–0.74 = good, 0.75–1.00 = excellent. Other methods such as area under the receiver operating characteristic curve (AUROC) were also reported [23, 24].

In general, *validity* refers to the degree to which a measurement tool captures the underlying construct of interest. We extracted all information regarding different forms of validity from included publications, the most commonly investigated being convergent validity (a specific subtype of construct validity), that examines how closely two instruments that are intended to measure the same construct are related. This is most often done by testing the correlation between the EQ-5D-5L and other measures of health or health-related quality of life (including those measuring pain, and mental or physical health or HRQoL). Other validity results extracted include known-groups validity (examining whether the 5L can distinguish between a priori determined groups).

*Responsiveness* is the ability of an instrument to capture true changes (e.g., due to a health intervention) in the construct of interest over time. Some argue that responsiveness is a subtype of validity or reliability [25]. Responsiveness is of particular importance for the EQ-5D-5L: one of the reasons the instrument was created was to address criticisms that the EQ-5D-3L was not sufficiently sensitive to change [26]. Responsiveness can be specific to population, context, and depends on the direction of change in the underlying construct [27]. In the case of the EQ-5D-5L, responsiveness addresses the question if the index value or individual items can detect relevant changes in underlying health. Preliminary research conducted on experimental five-level versions of the EQ-5D found its index value to be sensitive to change. Commonly used methods evaluating responsiveness include standardized effect size (SES) and/or standardized response mean (SRM) [25, 27, 28]. Both standardize the difference in means from two measurement points by dividing by standard deviation (of the mean or of the change scores). An SES of 0.2 to 0.3 is considered small, ≈ 0.5 medium and ≥ 0.8 large effect sizes [29]. Some studies examined the EQ-5D-5L’s ability to detect a change as defined by external criteria, or anchor, to estimate minimally important differences (MID) or the smallest change in score that is beneficial or relevant for patients [27, 28, 30]. The external anchor is usually a patient-assessment.

### Analysis

Due to the heterogeneity of studies and outcomes included, we were only able to summarize three outcomes across studies: proportion of respondents reporting the best health, mean index values, and EQ-5D-5L’s correlations with other measures (Spearman’s or Pearson’s Rho). When multiple index scores are reported in a study, the most up to date (EQ-5D-5L as opposed to the interim or ‘crosswalk’) or most appropriate (closest to the sampled population) index scores were extracted. The signs of correlation coefficients were changed if authors had not corrected for the directionality of the scales. Subgroup analysis was performed when there were at least three studies representing a relevant subgroup.

Data were pooled by means of random-effects models using inverse variance weight for pooling. Pooling was based on Fisher’s *z* transformation of correlation coefficients and logit transformation of proportions. Microsoft excel was used for data extraction, while R was used for data analysis [31]. The R package “meta” was used to estimate pooled values [32].

## Results

**Fig. 1:**
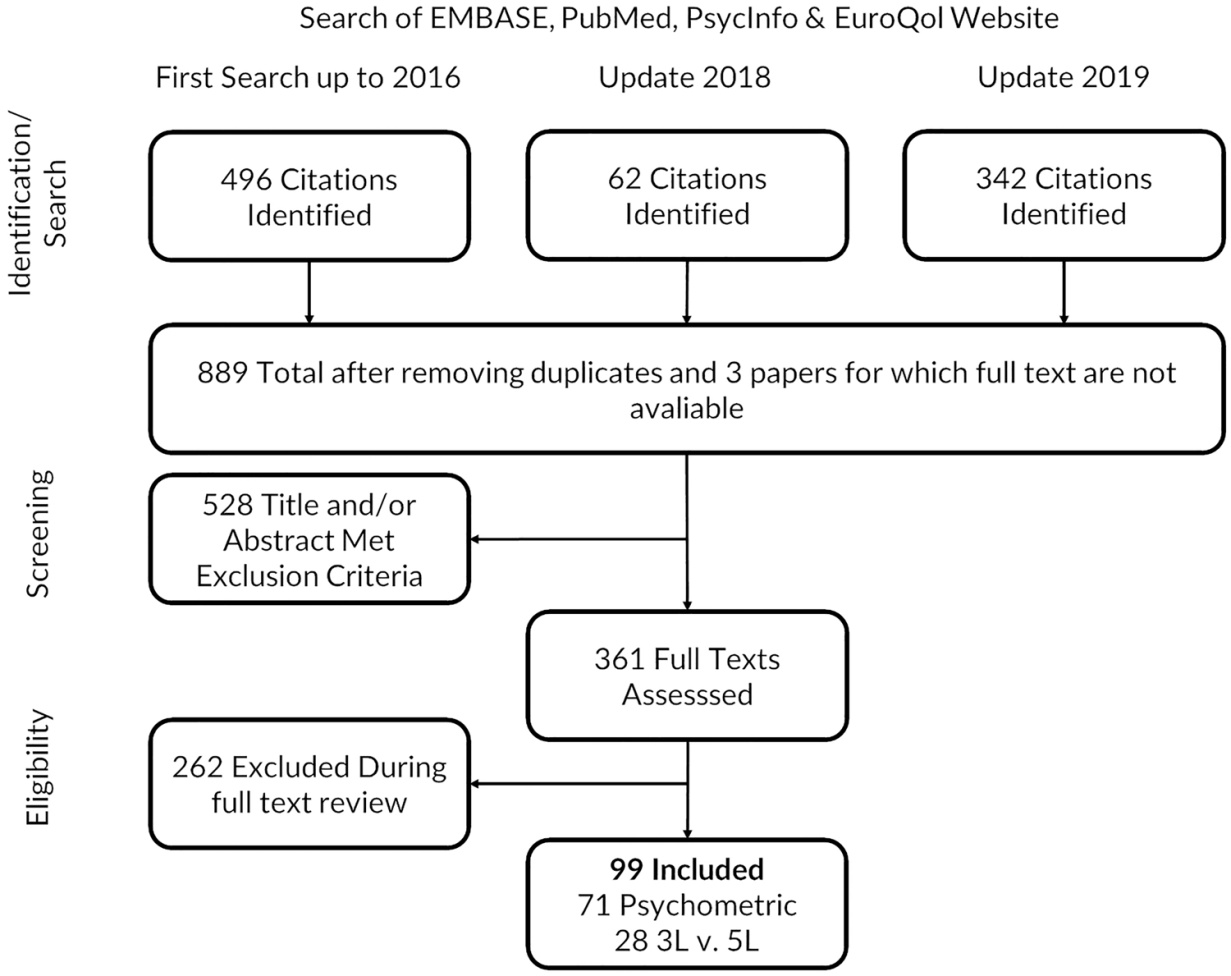
Literature search and inclusion/exclusion results

We identified 496 papers during the initial search and additional 397 papers during the updates in 2018 and 2019, of which 99 papers were included for review (Fig. 1; reference list A). These papers included general population (*n* = 32) and patients (*n* = 58) from 32 countries (see Table 1). The country where the most numerous studies were conducted was the UK/England (*n* = 18), while Canada, Germany, Singapore and the USA were the locations with the second most numerous studies (*n* = 8 each). The patient groups represented by the most studies are musculoskeletal/orthopedic (*n* = 8), cancer (*n* = 8) and lung/respiratory diseases (*n* = 7). The Multi-Instrument Comparison study (MIC) [A20, A26, A31, A36–A38, A49, A53, A77, A79, A96] and the study that developed a method of deriving 5L interim index values from 3L value sets [A4, A6, A83] were represented by 11 and 3 studies, respectively. General characteristics of included studies can be found in Supplementary Table 2.

**Table 1:** Psychometric properties of EQ-5D-5L

First author publication year [reference]	Country	Disease area/study population	Floor^a^ > 15%	Missing ≥ 5%	Test–retest ICC, Cohen’s kappa (*κ*)^c^	Known-group validity^b^
*Musculoskeletal diseases and orthopedic patients*						
Buchholz 2015 [A23]	GER	Orthopedic, psychosomatic, rheumatologic rehabilitation patients	No	No		
Conner-Spady 2015 [A24]	CA	Osteoarthritis, referred for total joint replacement	No	No	ICC Index: excellent Kappa: MO good; SC excellent; UA good; PD good; AD excellent	
Greene 2015 [A29]	USA	Patients undergoing total hip arthroplasty	No			
Whitehurst 2016 [A56]	CA	Spinal cord injury	MO & SC			
Bilbao 2018 [A68]	ESP	Hip or knee osteoarthritis	No	No		Statistically sign. difference across WOMAC scores and self-rated health
Cheung 2018 [A72]	CN	Patients attending a back pain clinic	No	No		Statistically sign. over disc degeneration and spinal surgery, but not other spine-related factors or pain
Conner-Spady 2018 [A73]	Manitoba, CA	Osteoarthritis; 1 year following total joint replacement	No		ICC: excellent	
*Diabetes*						
Pan 2014 [A34]	CN	Outpatients with type 2 diabetes mellitus	No			
Pattanaphesaj 2015 [A35]	TH	Diabetes; treated with insulin	No	No	ICC: good Kappa: MO fair; SC nr; UA fair; PD fair; AD fair	
Wang 2015 [A42]	SG	Type II diabetes	No			
Wang 2016 [A55]	SG	Diabetes	No			
McClure 2018 [A87]	CA	Type II diabetes	No			
*Cancer*						
Kim 2012 [A2]	Korea	Cancer patients receiving ambulatory chemotherapy	No		ICC: excellent Kappa: MO good; SC good; UA fair; PD fair; AD fair	
Lee 2013 [A9]	SG	Histologically confirmed breast cancer				Across Oncologist, Patient evaluated performance status, treatment mode, and evidence of disease
Kouwenberg 2019 [A98]	NL	Breast reconstruction/mastectomy patients				Across radiotherapy type, surgery group and age
*Skin diseases*						
Swinburn 2013 [A12]	UK	Psoriasis				Trending as expected across skin-specific questionnaires (DLQI and SAPAPSI)
Poor 2017 [A63]	HU	Psoriasis	No	No		Not sign. across age groups, but sign. across gender.
Yfantopoulos (2017a) [A64]	GR	Psoriasis	No			
Tamasi 2018 [A90]	HU	Pemphigus vulgaris and pemphigus foliaceus				Statistically sign. across severity of disease, symptoms and comorbidities. Not across gender or treatment status
*Stroke*						
Golicki 2015 [A27]	PL	Stroke		No		Trends as expected across age, modified Rankin Scale, Barthel Index, Stroke type
Golicki 2015 [A28]	PL	Stroke	MO, SC, UA at baseline	No		
Chen 2016 [A46]	Taiwan	Stroke	Only SC			
*Mental health diseases*						
Mihalopoulos 2014 [A20]	AU, UK, USA, CN, NOR, GER	People reporting depressive symptoms [MIC]^e^				Strongly across levels of depression
Camacho 2018 [A70]	UK	Mental health conditions	No			
Engel 2018 [A77]	AU, CA, GER, NOR, UK, USA	Depression [MIC]^e^				Statistically sign. between healthy and depressive samples: effect size is large
*Cardiovascular diseases*						
White 2015 [A43]	UK	Symptomatic cardiac arrhythmia before and after cardiac ablation			ICC: excellent	
Chuang 2019 [A94]	FR, AT, GER, I, ESP, CH, UK	Acute pulmonary embolism or deep vein thrombosis	No	No		Moderately across embolism types
Gao 2019 [A96]	AU, CA, GER, NOR, UK, US	Heart disease				Statistically sign. differences across age, gender, education and MacNew Heart Disease scores
*Lung diseases*						
Lin 2014 [A19]	USA	Chronic obstructive pulmonary disease				
Szentes 2018 [A89]	GER	Interstitial lung diseases		No		
Hernandez 2019 [A97]	UK, FR	Asthma				Moderately to strongly with medication use and asthma control
*Liver diseases*						
Scalone 2011 [A1]	I	Different severe chronic hepatic diseases	No	No		
Scalone 2013 [A10]	I	Chronic hepatic diseases		No		
Jia 2014 [A18]	CN	Inpatients with hepatitis B	No		ICC: Excellent Kappa: MO excellent; SC good; UA excellent; PD excellent; AD excellent	
*Blood diseases*						
Batt 2018 [A66]	USA	Hemophilia				Statistically sign. across age, employment, cohabitation, existence of chronic conditions and pain. Not sign. across education, BMI groups, cohabitation, Hemophilia severity, treatment type
Buckner 2018 [A69]	USA	Hemophilia B and caregivers of children (< 18 years) with hemophilia B				Statistically sign. differences across self-reported anxiety, depression, arthritis, pain, age, hemophilia severity, functional status
*Kidney diseases*						
Yang 2015 [A44]	SG	Diagnosis of End-stage renal disease on *Peritoneal* or *hemo*dialysis	No			Strongly across comorbidity categories and symptoms, but weakly across dialysis adequacy, hemoglobin levels and burden
Thaweethamcharoen 2018 [A91]	TH	Patients on peritoneal dialysis				
*Central nervous system diseases*						
Garcia-Gordillo 2014 [A16]	ESP	Parkinson’s disease	No			
Fan 2018 [A78]	UK	Parkinson’s Disease	No			
*Other patient types and studies that incorporate several disease groups*						
Tran 2012 [A3]	VN	Diagnosis of HIV/AIDS	No			
van Hout 2012 [A4] Janssen 2013 [A6]	DK, UK, NL, PL, I, SCO	Crosswalk study^d^	No	No		Moderately with age and smoking, not with education
Craig 2014 [15]	USA	Patients with chronic conditions from a national representative sample of adults	No			
Richardson 2015 [A37] Mitchell 2015 [A31]	AU, CA, GER, NOR, UK, USA	MIC study^e^				Strongly across different chronic disease groups vs. healthy
Sakthong 2015 [A39]	TH	Outpatient patients taking continuous medication at least 3 months for 14 disease groups	No		ICC index: excellent Kappa: MO good; SC fair; UA fair; PD fair; AD fair	Statistically sign. across age, gender, education, employment, self-rated health, comorbidities, number of medicines and perception of disease control
Lamu 2016 [A49]	AU, CA, GER, NOR, UK, USA	MIC study^e^				Weakly with subjective well-being
Rogers 2016 [A54]	UK	Deaf persons using British sign language	No		ICC: excellent Kappa: MO good; SC fair; UA fair; PD good; AD fair	Weakly to moderately with CORE 10, CORE 6D
Fermont 2017 [A59]	UK	Severe and complex obesity; undergoing bariatric surgery				Not statistically sign. across BMI levels (Above and under 50) or those with comorbidities versus those without.
Bewick 2018 [A67]	UK	Chronic rhino sinusitis patients	No			
Easton 2018 [A75]	AU	Older residents of care facilities with dementia or cognitive impairments and proxies				Moderate to small differences across cognition scores and modified Barthel index categories
Janssen 2018 [A83]	PL, DK, England, I, SCO, NL	Crosswalk study^d^	Only UA			
Kohler 2018 [A84]	India	Post vaginal birth or cesarean section	MO, SC, UA only at baseline			
Gandhi 2019 [A95]	SG	Cataract surgery	No			
Rencz 2019 [A99]	HU	Crohn’s disease	No	No		Statistically sign. differences across age groups and chronic conditions
*General population*						
Kim 2013 [A8]	KOR	Nationally representative general population			ICC: excellent Kappa: MO good; SC poor; UA good; PD fair; AD poor	
Agborsangaya 2014 [A13]	CA	General population	No	No		
Hinz 2014 [A17]	GER	General population	No			
Feng 2015 [A25]	England	General population	No			
Mulhern 2015 [A32]	UK (Yorkshire)	General adult population	No			
Scalone 2015 [A40]	I	General population; quota sampling	No			
Augustovski 2016 [A45]	Uruguay	General population; sampling quotas by location	No			
Ferreira 2016 [A47]	Portugal	Students from 2 universities aged 30 years or under	No	No		Statically sign. across gender, health condition, labor situation, marital status
McCaffrey 2016 [A50]	AU	South Australian general population	No			
Oremus 2016 [A52]	CA	Toronto area general population	No			
Huber 2017 [A60]	GER	General population	No			
Konnopka 2017 [A61]	GER	General population				Each dimension sign. distinguished between categories of “dimension-specific” indicators; Index statistically sign. across age, education, diseases but not marital status
Nguyen 2017 [A62]	VN (Hanoi)	Randomly selected resident adults of the city of Hanoi	No			Sign. across age, occupation, education, income, symptoms, chronic conditions; not Over health services usage
Yfantopoulos 2017b [A65]	GR	General middle-aged and elderly population	No	No		Statistically sign. across age, gender and smoking status
Purba 2018 [A88]	Indonesia	Indonesian representative population	No		Assessed Gwet’s AC, acceptable for dimensions. ICC low (0.37) for index	Statistically sign. across age, ethnicity and gender, but not across residence, education, income or religion.
Hernandez 2018 [A81]	ESP	Spanish National Health Survey 2011–2012	No			
Marti-Pastor 2018 [A86]	ESP	Representative general population	No			
Ge 2019 [A80]	SG	Young (21–44 years), middle-aged (45–64 years), older adults (≥ 65 years)				
*Proxies*						
Bhadhuri 2017 [A57]	UK	Family members of meningitis survivors				

*Sign.* significant(ly)

*AU* Australia, *AT* Austria, *CA* Canada, *CH* Switzerland, *CN* China, *DK* Demark, *ESP* Spain, *FR* France, *GER* Germany, *GR* Greece, *HU* Hungary, *I* Italy, *KOR* South Korea, *NL* Netherlands, *NOR* Norway, *PL* Poland, *SCO* Scotland, *SG* Singapore, *TH* Thailand, *UK* United Kingdom, *USA* United States of America, *VN* Vietnam

Blank cells imply that the study did not investigate and/or report on the psychometric property

^a^Floor defined as reporting worst health response levels 5 (“extreme problems” or “unable to”) for EQ-5D-5L items (Mobility MO, Self-Care SC, Usual Activities UA, Pain/Discomfort PD, Anxiety/Depression AD) and on the profile (‘55555’). When not specified, reports of the worst health level for all dimensions and the profile were below 15%

^b^Generally assessed with effect size or tests of difference in means

^c^Kappa and ICC defined as [22]: (1) < 0.40 = poor. (2) 0.40–0.59 = fair. (3) 0.60–0.74 = good. (4) 0.75–1.00 = excellent

^d^Crosswalk study include: chronic obstructive pulmonary disease/asthma, diabetes, liver disease, (rheumatoid) arthritis, cardiovascular disease, stroke, depression, personality disorders, students

^e^Multiple Instrument Comparison (MIC) study includes: arthritis, asthma, cancer, depression, diabetes, hearing loss, heart disease (from AU, CA, GER, NOR, UK, USA)

**Table 2:** Responsiveness of EQ-5D-5L index values

First author year [reference]	Patient/population group	Value set	Improved		Stable		Deteriorated		All	
			SES	SRM	SES	SRM	SES	SRM	SES	SRM
*Studies using a value set for the 3L version of the EQ*-*5D/interim scoring method*										
Lee 2013 [A9]	Singaporean breast cancer patients at baseline and 1 week later: regressed with self-reported performance status	Interim scoring method [A5] Japanese 3L value set							0.54^b^	
	Singaporean breast cancer patients at baseline and 1 week later: regressed with self-reported quality of life								0.69^b^	
Swan 2013 [A11]	Patients before and after colonoscopy screening	Interim scoring method [A5] Unclear which 3L value set was used							0.50	0.44
Jia 2014 [A18]	Hepatitis B patients at baseline and 1 week after	Interim scoring method [A5] Chinese 3L value set	Absolute increase of 0.029–0.073 for index values for the subsample of patients with improved health. There is not enough information to calculate the SES.							
Golicki 2015 [A28]	Stoke patients initial hospitalization and 4 mo after therapy: mRS-based criterion	Interim scoring method [A5] Polish 3L value set	0.51	0.69			− 0.25	− 0.25		
	Stoke patients initial hospitalization and 4 mo after therapy: Barthel index-based criterion		0.71	0.86			− 0.40	− 0.47		
Chen 2016 [A46]	Stroke patients before and 3 to 4 weeks after therapy	Interim scoring method [A5] Japanese 3L value set							0.40	0.63
Conner-Spady 2018 [A73]	Pre to 1 year post TJR (hip)	Interim scoring method [A5] UK 3L value set							1.86	1.53
	Pre to 1 year post TJR (knee)								1.19	1.04
Kohler 2018 [A84]	Vaginal birth 3 to 7 days postpartum	Interim scoring method [A5] UK 3L value set							0.78^a^	
	Vaginal birth 21 to 30 days postpartum								1.18^a^	
	Cesarean Sect. 3 to 7 days postpartum								0.90^a^	
	Cesarean Sect. 21 to 30 days postpartum								1.65^a^	
Gandhi 2019 [A95]	Before and after cataract surgery	Interim scoring method [A5] Singaporean & English 3L value sets							0.25	0.23
	Before and after cataract surgery								0.26	0.23
*Studies using a value set for the 5L version of the EQ*-*5D*										
Sakthong 2015 [A39]	Patients of university hospitals 1 to 2 weeks apart	Thai 5L value set	0.33				− 0.29			
Nolan 2016 [A51]	COPD outpatients before and 8 weeks after pulmonary rehabilitation	English 5L value set							0.27^a^	
Fermont 2017 [A59]	Patients with severe/complex obesity before and 6 mo after bariatric surgery	English 5L value set	0.25	0.30^a^	− 0.08	− 0.09^a^			0.16	0.19
Bilbao 2018 [A68]	Patients with hip or knee osteoarthritis from hospital/clinic visit and 6 mo after	Spanish 5L value set	0.40	0.38	0.05	0.06	0.39	0.42		
Campbell 2018 [A71]	3 mo after bariatric surgery	English 5L value set							0.40^a^	
	1 year after bariatric surgery								0.32^a^	
McClure 2018 [A87]	Baseline to 1 year after: longitudinal study of diabetes patients	Canadian 5L value set	0.20	0.31			0.29	0.44		
Wijnen 2018 [A93]	Epilepsy patients pre intervention program to 12 mo after	Dutch and English 5L value sets							−0.017	−0.023
Chuang 2019 [A94]	Baseline to 1 year after: longitudinal study of venous thromboembolism patients	English 5L value set							0.44	
	Baseline to 1 year after: longitudinal study of venous thromboembolism patients								0.55	
*Studies not reporting which value set was used*										
White 2016 [A43]	UK patients with cardiac arrhythmias pre and 8–16 weeks post catheter ablation	Not reported							− 0.22	− 0.29
Bhadhuri 2017 [A57]	Non-carers of meningitis survivors 1 year apart	Not reported	0.01		− 0.19		− 0.14			
	Carers of meningitis survivors 1 year apart		0.19		− 0.02		− 0.27			
	Carers with fewer hours of care of meningitis survivors 1 year apart		− 0.16		0.05		− 0.31			

When papers reported multiple results for responsiveness, the SES and SRM are reported in this table for comparability. *SES* standardized effect size, *SRM* standardized response mean, *QoL* quality of life, *yr* year, *mo* month, *TJR* total joint replacement

^a^Effect size was calculated from available information in the paper

^b^Paper calculated effect size using regression methods: $\frac{\text{Regression}\; \text{coefficient}}{\text{residual}\; \text{standard}\; \text{deviation}}$

### Distribution properties

**Fig. 2:**
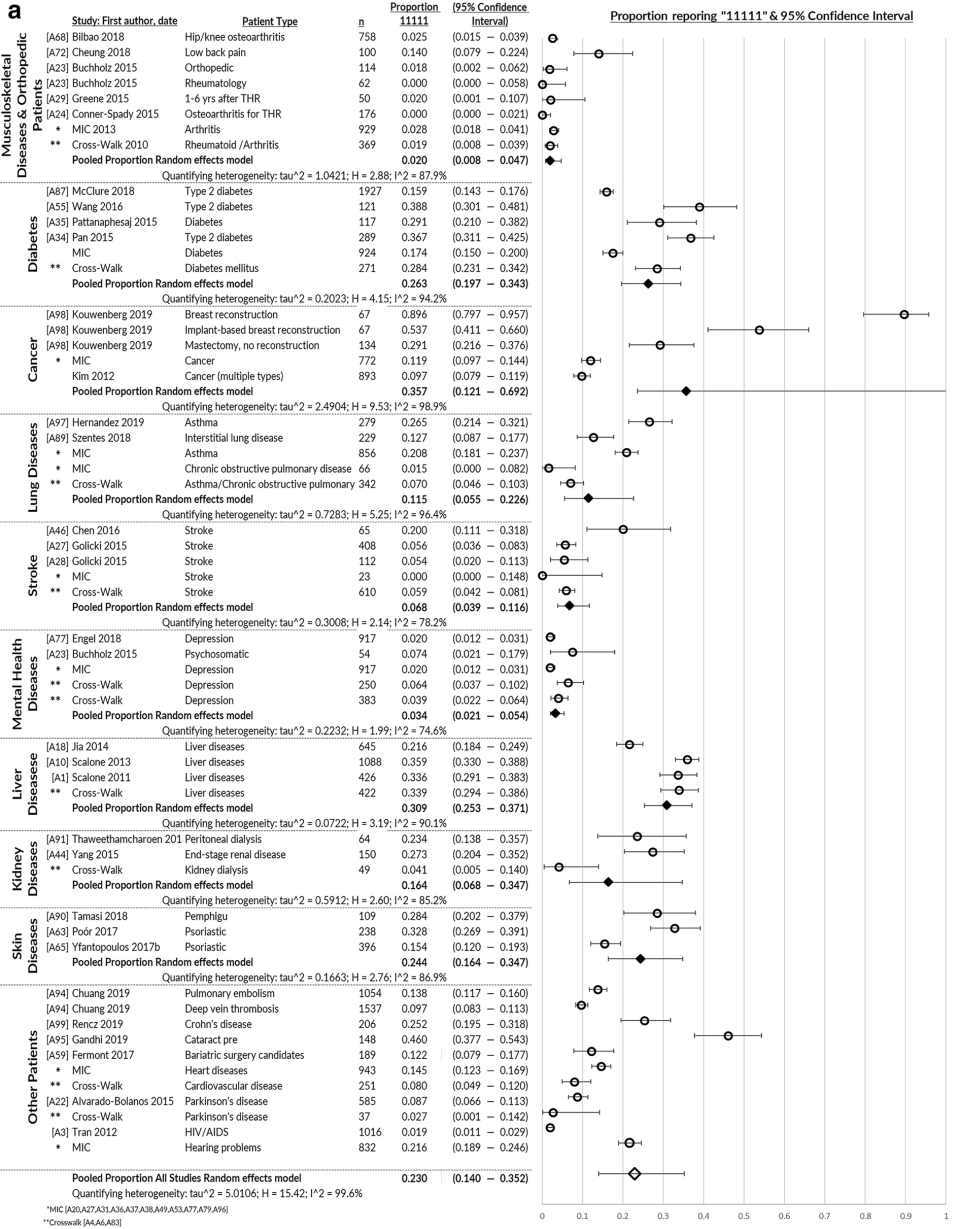

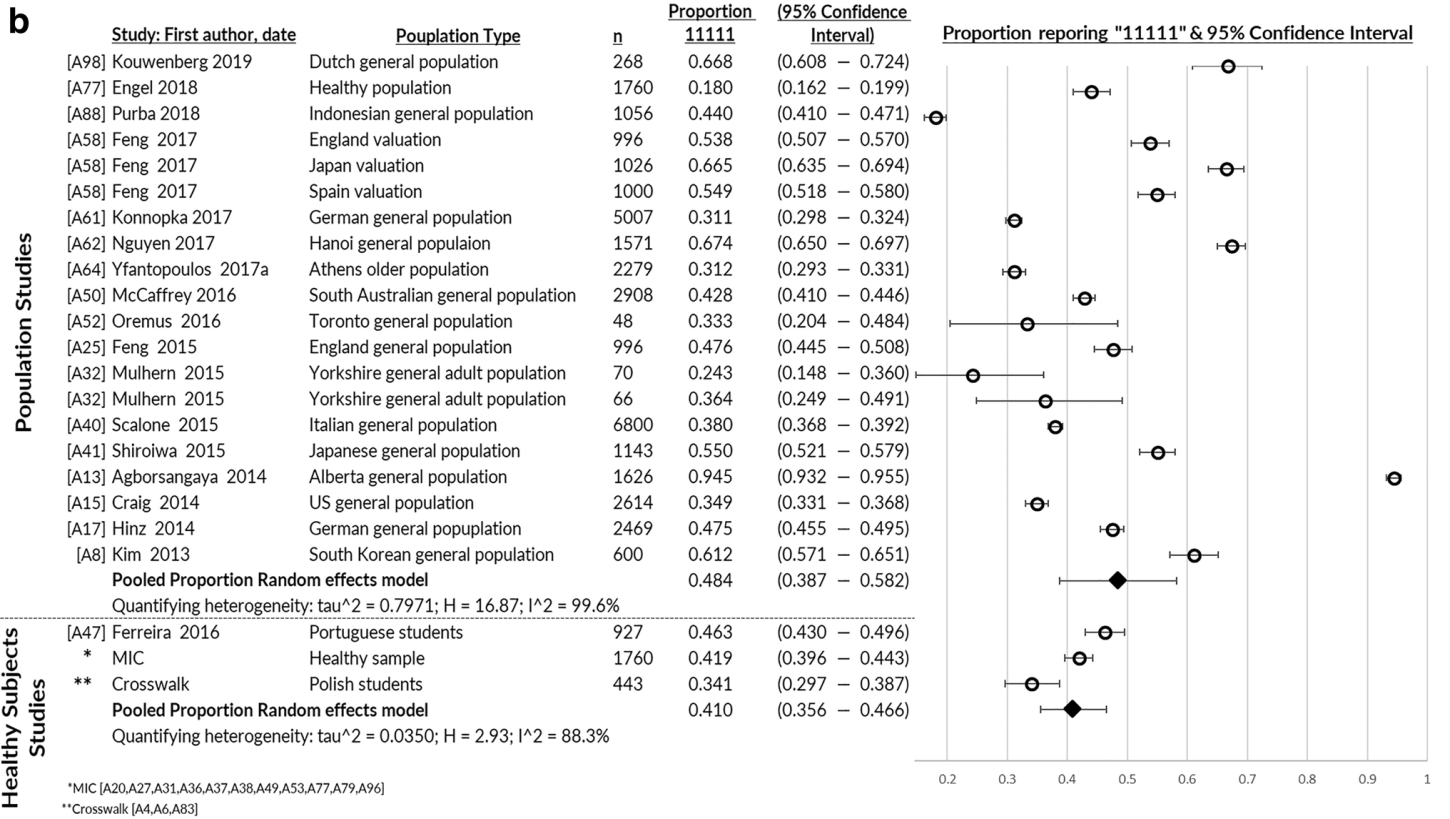
**a** Proportion reporting no problems on the EQ-5D-5L profile “11111”: pooled across health conditions. **b** Proportion reporting no problems on the EQ-5D-5L profile “11111”: pooled for general and healthy populations

Missing values (17 of 17 papers) and most severe health state (43 of 48 papers) were under 5% and 15%, respectively, showing the 5L to be feasible and free from floor effects (Table 1). Studies with greater than 15% reporting the most severe health (in certain dimensions) were those studying patients with stroke [A28, A46], spinal cord injury [A56], women just after giving birth [A84] and patients with chronic illnesses [A83]. These patients were reporting severe health impairments in MO, SC, and/or UA. Enough information was reported by 48 studies to pool proportion reporting the best health state ‘11111,’ which was 23% for patients, ranging from 2% (musculoskeletal diseases) to 36% (cancer; Fig. 2a). Pooled proportion of over 15% at full health was observed for patients with diabetes, cancer, liver diseases, kidney diseases and skin diseases. General and healthy population studies were 48% and 41% reporting full health, respectively (Fig. 2b).

By dimension, proportions reporting “no problems” were smallest across the board for stroke, while SC consistently had large ceilings except for patients with stroke, diseases of the nervous system and diseases of the musculoskeletal system (pooled proportion reporting “no problems” in EQ-5D-5L dimensions can be found in Supplementary Table 3). Konnopka and Koenig (2017) also found SC to be most problematic in terms of percentage at the ceiling, even for those reporting four or more diseases and needing one or more hours of daily care [A61].

**Fig. 3:**
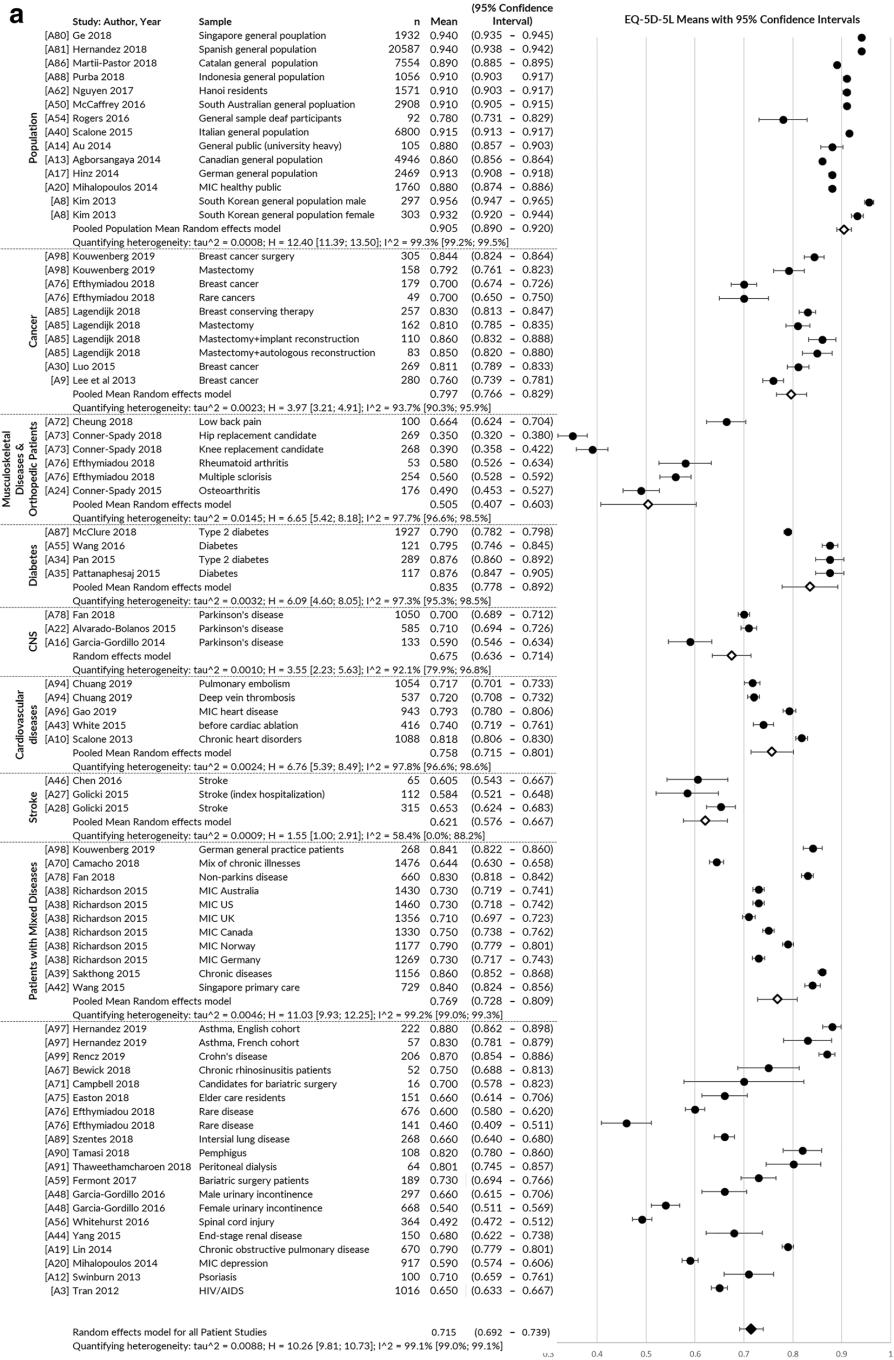

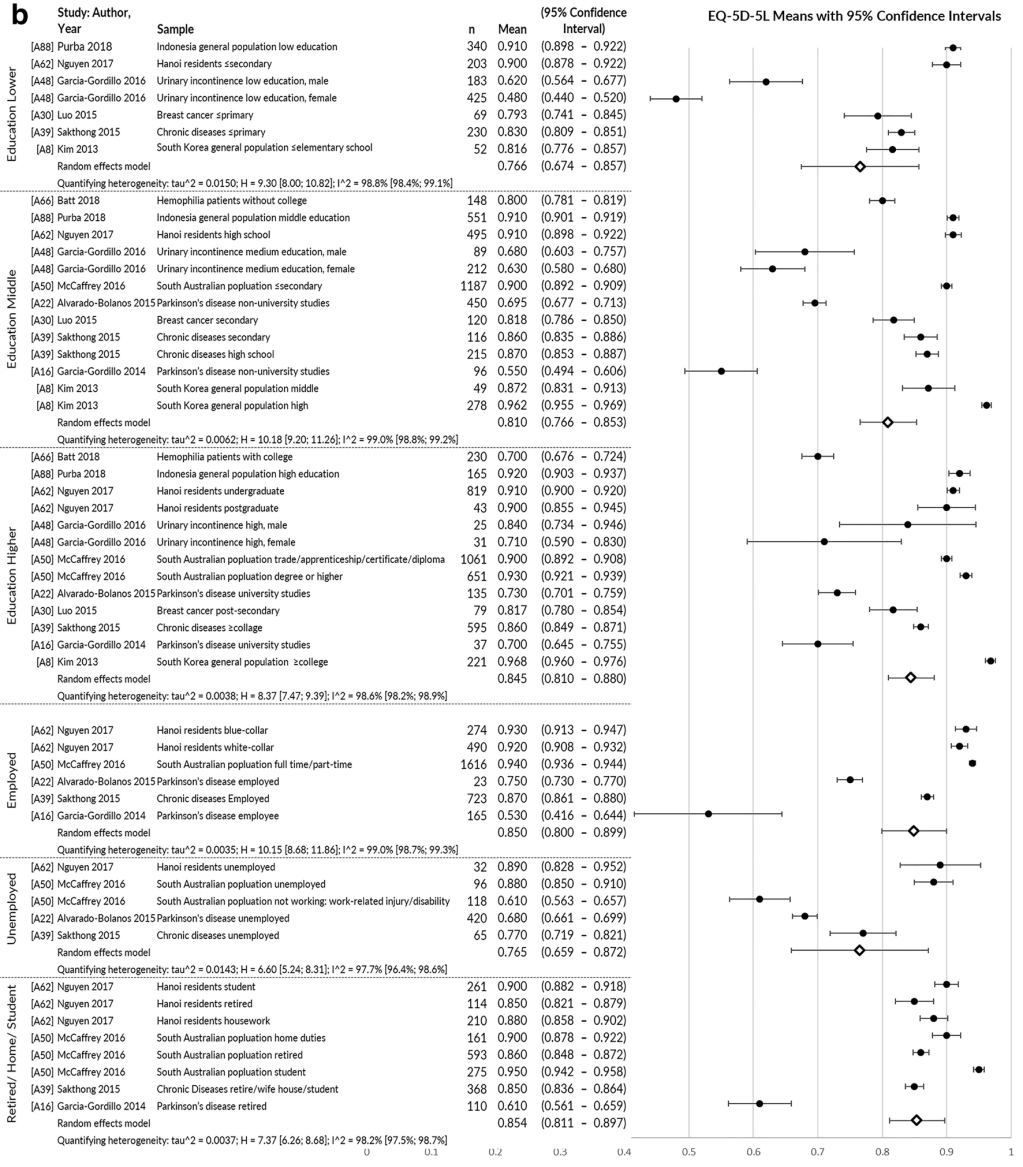
**a** EQ-5D-5L index value mean: pooled across health conditions. **b** EQ-5D-5L index value mean: pooled across education level and employment status

Index value means could be pooled from 58 publications, showing they were generally lower for disease groups than healthy populations and lower socio-economic/socio-demographic groups than higher (Fig. 3a, b).

### Reliability

Nine papers addressed test–retest reliability, eight found the scale agreement (ICC) excellent and the remaining study finding an ICC of 0.7. However, five studies found fair agreement on the item level (Cohen’s Kappa) for certain dimensions: they tend to be smaller for PD and highest for MO (Table 1).

### Validity

Studies examining *construct validity* typically compared the EQ-5D-5L to the EQ-5D-3L: the focus has been on the response categories as the items themselves were identical. As we did not include studies with experimental versions of the 5L, most of the earlier studies examining the construct validity of various response options of the 5L have not been included. One included study used exploratory factor analysis to examine the structure of the EQ-5D-5L, Satisfaction with Life Scale and MacNew questionnaire [A96]. They found MO, SC, UA, and PD to load onto one factor with other physical health and usual activity items, and AD to load onto a second factor including items addressing mood, depression, and confidence. Of the five included papers addressing *content validity*, three used qualitative methods. Keeley et al. (2013) sampled research professionals who found the SC item to be too narrowly defined and the UA item to be too broad, while deeming PD and AD as the most relevant dimensions related to health-related quality of life [A7]. Whitehurst et al. (2014) sampled patients with spinal cord injuries, who generally found the 5L to be relevant for their health problems [A21]. However, some found the instrument to lack coverage of specific aspects of spinal cord injury. A more recent qualitative study found the EQ-5D-5L to lack relevancy for asthma patients except for some physical limitations, but also praised the instrument for its generic nature [A92].

Craig et al. (2014) found via regression analysis that the 5L encompasses a slightly larger range of EQ-VAS scores from best to worst health state compared to the 3L [A15]. Janssen et al. 2018 also investigated the distance between the 3L and 5L levels using a direct approach asking patients to place the labels onto a horizontal VAS scale, finding a larger range covered by the 5L [A83].

**Fig. 4:**
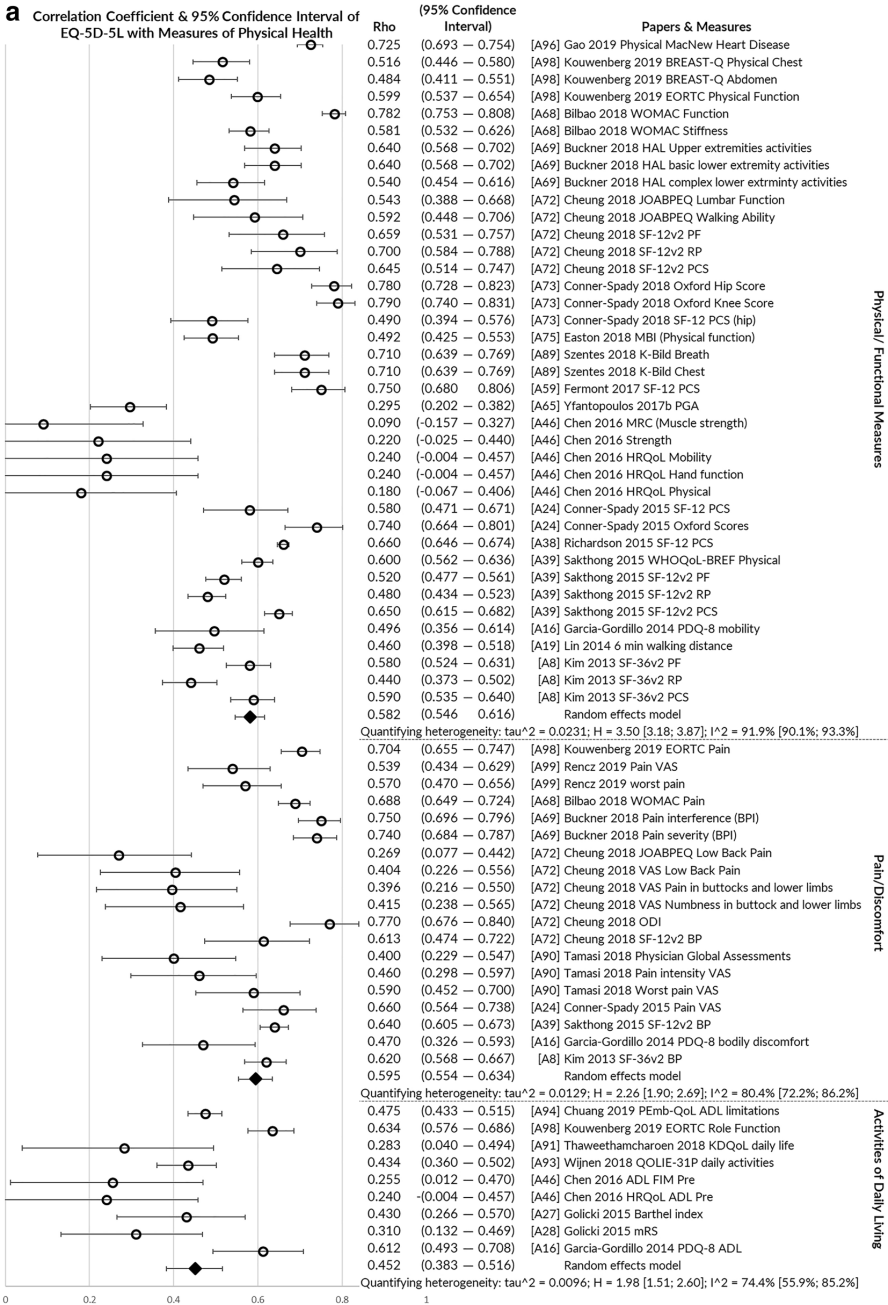

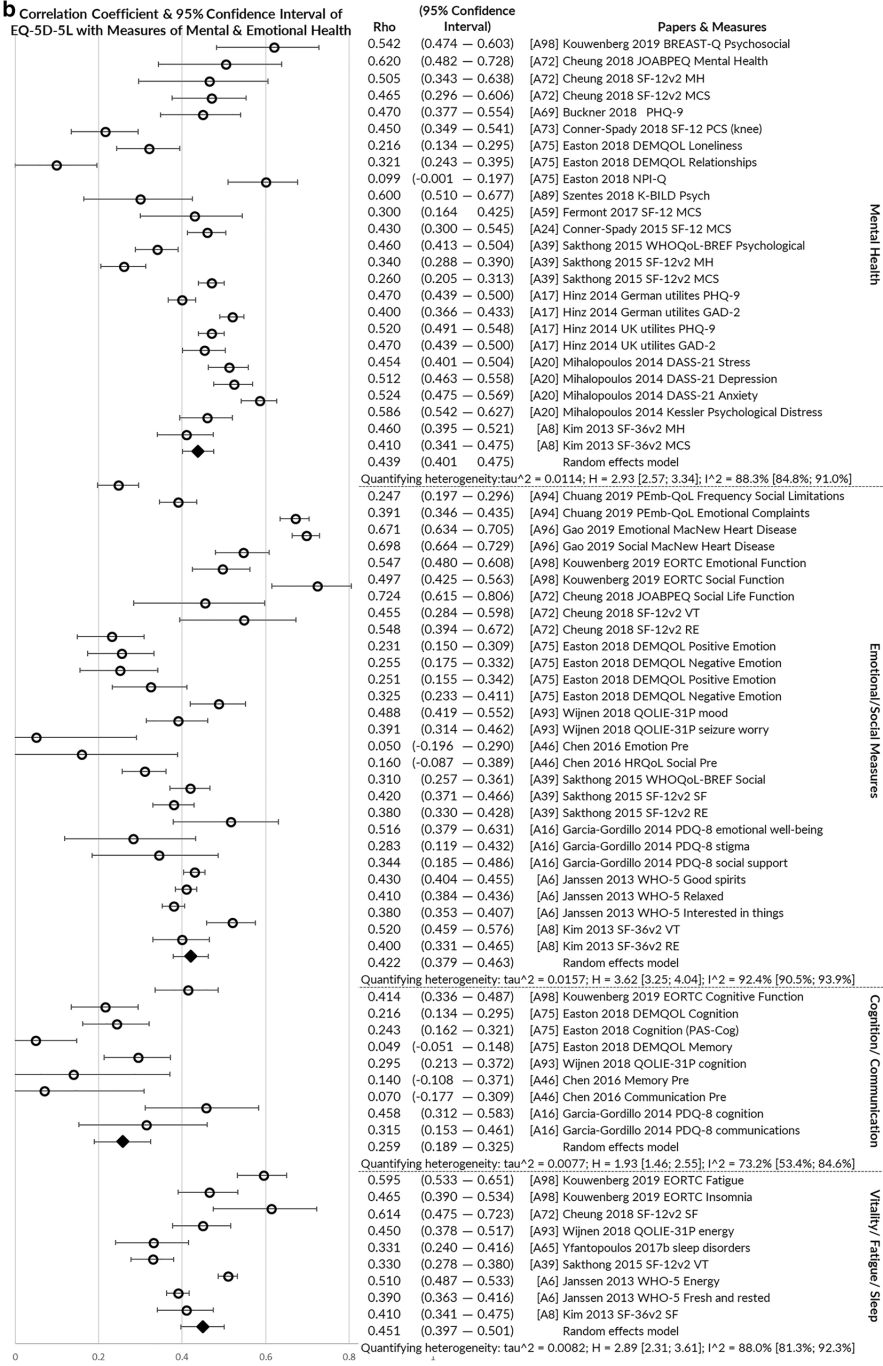

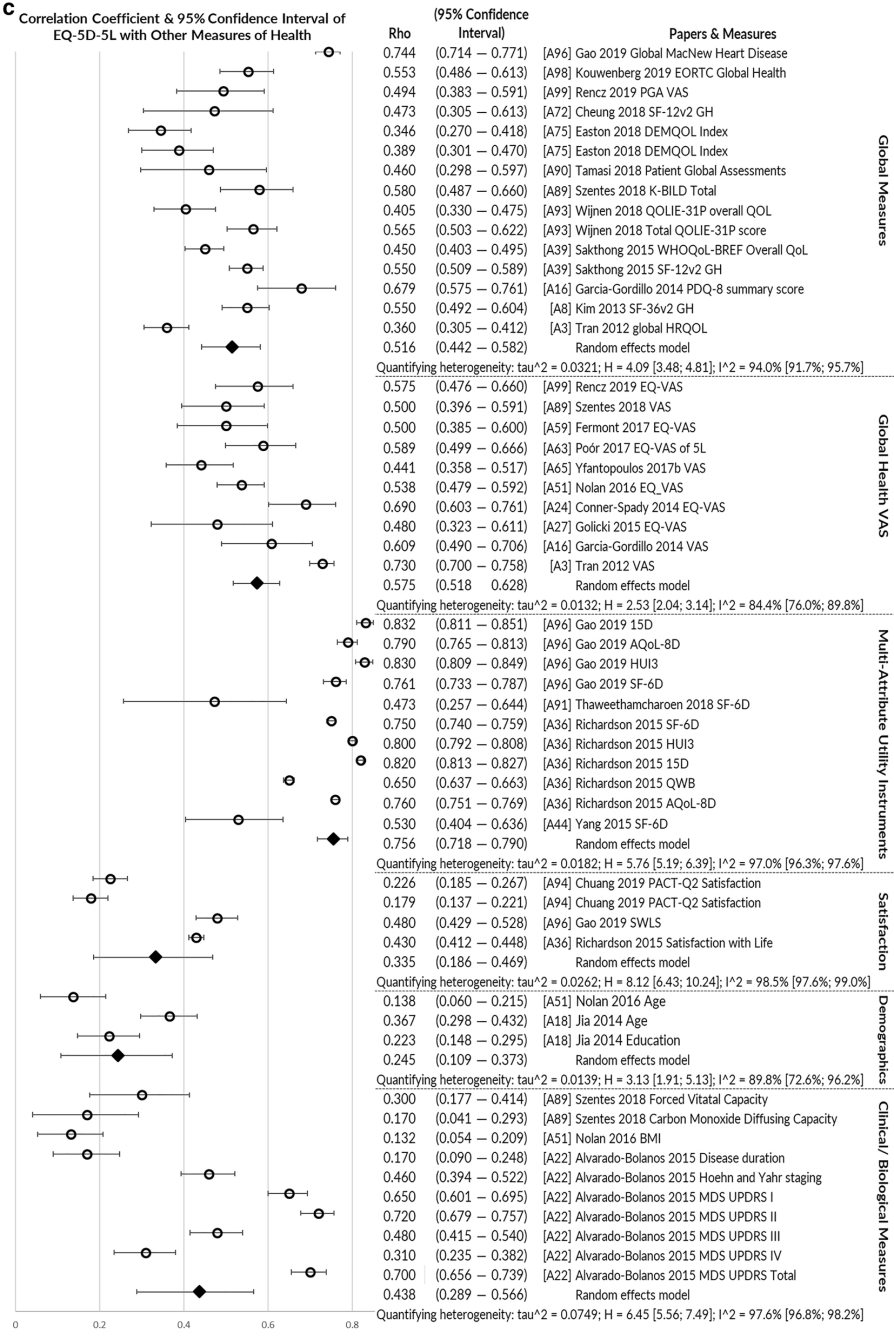
**a** Pooled correlation coefficient for EQ-5D-5L index value with other physical health measures. **b** Pooled correlation coefficient for EQ-5D-5L index value with other mental, emotional, cognitive and fatigue/vitality health measures. **c** Pooled correlation coefficient for EQ-5D-5L index value with other global health, clinical and non-health measures

*Convergent validity* was assessed by the greatest number of papers (*n* = 33), usually examining correlations of EQ-5D-5L with other measures of health using Pearson’s correlation or Spearman’s Rho rank correlation coefficient. Figure 4a–c illustrates pooled correlations of the EQ-5D-5L index value with other measures of physical health, mental/social/cognitive health and global health. The strongest correlations were observed for multi-attribute utility instruments (pooled rho = 0.756), physical/functional measures (pooled rho = 0.582) and pain/discomfort measures (pooled rho = 0.595). The EQ-5D-5L index value correlated poorly with measures of satisfaction (pooled rho = 0.335) and cognition/communication (pooled rho = 0.259).

On a dimension level, the strongest correlation was observed for PD and pain measures (pooled rho = 0.636), while all items correlated poorly with measures of cognition/communication and vitality/fatigue/sleep. AD was the only item to show (moderate) correlation with mental (pooled rho = 0.461), emotional and social health items (pooled rho = 0.413). Pooled correlation of EQ-5D-5L dimensions and other measures of health can be found in Supplementary Table 4.

Bhadhuri et al. 2017 examined the EQ-5D-5L’s ability to measure spillover effects and found strong correlations between EQ-5D-5L scores of family of meningitis survivors and survivors’ social lives (Spearman’s Rho = 0.52, 0.45), exercise (rho = 0.55, 0.82), and personal health (rho = 0.88, 0.95) [A57]. Poor correlations were found between carers’ and survivors’ EQ-5D-5L dimensions (rho = 0.07 to 0.24), index (rho = 0.19, 0.26), and EQ-VAS (rho = 0.22, 0.24).

Table 2 includes information from studies, which examined *validity other than convergent*. Generally, the 5L can distinguish across disease groups, disease severity, symptoms, and related groups, and also across age and education. However, it does not consistently distinguish across groups differing with certain clinical outcomes (e.g., presence of deformities in the spine, frequency of medication use, gender, use of health services, and marital status.

### Responsiveness

Fifteen studies examined whether the EQ-5D-5L captures change in health over time. All of these papers included SES and/or SRM. Although not reported, the SES could be calculated for two papers using reported information [A71, A84]. Five assessed results across respondents who improved, remained stable or deteriorated over time based on an anchor measure [A28, A39, A57, A59, A68, A87]. Four papers also reported MID [A46, A50, A71, A85]. Two used retrospective items to define change [A50, A71]. Table 4 summarizes the responsiveness results—when available, the SES and SRM are used for ease of interpretability. The EQ-5D-5L index values typically had moderate effect sizes for improved patients and those expected to improve (over the course of medical or therapeutic intervention). The largest effect sizes were observed for patients days and weeks after giving birth [A84]. Compared to other instruments, the 5L generally performs as well or better. Two additional papers addressed dimension-level changes [A23, A74], both finding the 5L to be more sensitive than the 3L. Crick et al. 2018 examined only the AD dimension and noted that both the 3L and 5L were limited in responsiveness [A74].

## Discussion

The EQ-5D is a generic preference-based health status instrument that has enjoyed widespread use since its creation in the 1980s [33]. The psychometric properties of the three-level version have been well established [34–40]. Any reluctance of using the more recently developed five-level version might come in part from limited experience and evidence for validity, reliability or responsiveness in different populations [41]. This review summarized published evidence on the psychometric properties of the EQ-5D-5L, which has been investigated in a broad array of countries, populations and contexts in the past decade. No studies found missing values to be problematic for the instrument, demonstrating feasibility. Test–retest results show potential problems with stability over time on an item level, but not at the instrument (index score) level. Note that internal consistency is not a relevant psychometric property for the EQ-5D-5L since its index score is based on a completely different measurement framework (as a preference-based measure).

Rather large proportions of respondents reporting the best health profile were observed for general population studies but less so for patient populations. The EQ-5D was conceptualized to measure deviations from full health (or negative health) and is more prone to larger ceilings than instruments that include positive health dimensions (e.g., the SF-6D). Therefore, studies with samples for which impact on the functions covered by the EQ-5D-5L (e.g., recovered cancer patients, liver disease, diabetes) is less relevant, other disease-specific instruments should be used in conjunction. On the item level, most studies, even those with populations in poorer health, reported a substantial ceiling with the dimension “self-care”, although the ceiling for self-care was low for respondents who were expected to have limitations with this function (e.g., patients before hip replacement surgery, patients shortly after cesarean section, patients with spinal cord injury [A21, A24, A84]). These results suggest that while most populations may not report problems in “self-care”, it is relevant for particular patient groups.

Our results overall solidly establish the validity of the EQ-5D-5L as supported by observed trends across subgroups (pooled means, known-group validity) as well as the convergent validity (correlation of items and index to other measures of health-related quality of life). Index values as well as the dimensions show moderate to strong correlations with physical/functional measures, pain, measures of mental and emotional health, activities of daily living and clinical/biological measures as well as with other multi-attribute utility measures. On the other hand, the 5L is not found to be correlated with satisfaction with life and cognition/communication measures. Indeed, current efforts investigating adding dimensions (so-called “bolt-ons”) to the 5L has identified cognition as an important dimension missing from the EQ-5D [42–44].

Included studies on responsiveness are heterogeneous in terms of the population, whether and which anchors were used, whether a health intervention was administered, and stratification of results across subgroups. This is not a problem unique to the EQ-5D-5L as, unlike other psychometric properties, there is not a set of recommended analyses to address responsiveness [25, 30]. Therefore, it is difficult to elucidate whether the EQ-5D-5L has problems with sensitivity to change in certain populations or with certain treatments. Despite this limitation, responsiveness is found to be acceptable by all included studies. A previous review found the EQ-5D-5L to be responsive to half of the conditions included, but found mixed evidence for the other half [26]. Responsiveness and sensitivity to changes in health is clearly an area that needs further investigation. Future studies could benefit from defining what a relevant change is for the EQ-5D-5L (MID) and defining appropriate anchor measures that can be used across populations (e.g., a level of change in EQ-VAS scores or a single self-rated health item). Parkin and colleagues (2016) demonstrated the EQ-5D-5L distribution to be affected both by the descriptive system and the value set applied [45]. Although not a focus of this study, the valuation method and applied utility scores are as important as the descriptive system when assessing responsiveness of index values. It has been shown that choice of value set has an impact on utility scores [46–49] and may change results of cost-utility analyses [48, 50, 51]. Other results show that the effect of value sets on utility scores is relatively small [A37, A83]. Due to the heterogeneity of studies found in this review, we have insufficient information to evaluate how value sets impact responsiveness. Future research will benefit from systematically examining responsiveness of the descriptive system and how choice of value set farther impacts responsiveness.

This review included nearly one hundred studies published in the past decade that investigated the psychometric properties of the EQ-5D-5L, the majority of which sample populations from western Europe, OECD countries and secondarily, from East Asia. This clearly reflects where the EQ-5D-5L is currently used [52]. However, almost a third of new user registrations in 2018 come from countries accounting for less than 1.5% of total registrations, demonstrating widespread as opposed to concentrated use of the instrument [52]. For instance, two reviews report rapid uptake of the instrument in Eastern Europe [53, 54]. Establishing validity in other regions is crucial as the EQ-5D-5L expands in its use. Similarly, as the EQ-5D instrument has expanded in its application, it would also be important to assess how well it performs in particular settings and applications, such as used to inform clinical practice, in health services research or in health surveillance programs.

## Study limitations

A limitation of this study is that studies using experimental versions of the EQ-5D-5L were excluded. Early experimental work on the content validity of the instrument [55–62] and investigations of bolt-on items [63] are therefore not captured by this review. Similarly, due to the very large number and range of quality of studies identified, we did not include application studies of the EQ-5D-5L which did not explicitly address psychometric properties, and therefore are missing distributional and perhaps responsiveness information that may have been captured by those publications. As already discussed, choice of value set and valuation methodology are as important as the descriptive system in the case of the EQ-5D. This review does not address valuation methods and therefore does not tackle a crucial component of the instrument and its index value. A previous review of valuation methodology provides valuable information on this topic [64].

## Conclusions

The EQ-5D-5L is a reliable and valid generic instrument that describes health status which can be applied to a broad range of populations and settings. The assessment of responsiveness, in particular, needs further and more rigorous exploration. Rather large ceilings persist in general population samples, reflecting the conceptualization of the EQ-5D instrument, which focuses on limitations in function and symptoms, and does not include positive aspects of health such as energy or well-being.

## Electronic supplementary material

Below is the link to the electronic supplementary material.

Supplementary material 1 (DOCX 65 kb)

## Abbreviations

15D: 15 measure of health-related quality of life
ADL: Activities of Daily Living
AQoL-8D: Assessment of Quality of Life (AQoL)-8D Multi-Attribute Utility Instrument
BMI: Body Mass Index
BREAST-Q: Breast surgery-specific patient-reported outcome measure
DASS-21: Depression, Anxiety and Stress Scale-21 Items
DEMQOL: Dementia Quality Of Life Questionnaire
GAD: Generalized Anxiety Disorder Scale
JOABPEQ: Japanese Orthopedic Association (JOA) Back Pain Evaluation Questionnaire
EORTC: European Organization for Research and Treatment of Cancer
EQ-VAS: Visual Analog Scale of the European Quality of Life-5 Dimensions (EQ-5D)
FIM: Functional Independence Measure
HAL: Hemophilia Activities List
HUI3: Health Utilities Index Mark 3
K-BILD: King’s Brief Interstitial Lung Disease Questionnaire
KDQoL: Kidney Disease Quality of Life Questionnaire
MBI: Modified Barthel Index
MDS UPDRS: Movement Disorder Society Unified Parkinson’s Disease Rating Scale (UPDRS)
MRC: Medical Research Council scales for muscle strength
mRS: Modified Rankin Scale
NPI-Q: Neuropsychiatric Inventory Questionnaire
ODI: Oswestry Disability Index
PACT-Q2: Perception of Anticoagulant Treatment Questionnaire (PACT-Q) Part 2
PHQ-9: Patient Health Questionnaire-9 Items
PEmb-QoL: Pulmonary Embolism Quality Of Life Questionnaire
PGA: Patient Global Assessment
QOLIE-31P: Quality of Life in Epilepsy-Patients-Weighted 31p
PAS-cog: Psychogeriatric Assessment Scale-Cognitive Impairment
QWB: Quality of Well-Being
SF-6D: Short Form-6 Dimensions
SF-12(v2): Short Form-12 Items Health Survey; v2—version 2 (Subscales: BP – Bodily Pain, GH – General Health, MH – Mental Health, PF, RE – Role Emotion, RP – Role Physical, SF – social functioning, VT – Vitality, Summary Scores: MCS – Mental Component Score, PCS – Physical Component Score)
SF-36(v2): Short Form-36 Items Health Survey; v2—version 2 (Subscales: BP – Bodily Pain, GH – General Health, MH – Mental Health, PF, RE – Role Emotion, RP – Role Physical, SF – social functioning, VT – Vitality, Summary Scores: MCS – Mental Component Score, PCS – Physical Component Score)
SWLS: Satisfaction with Life Scale
WHO-5: World Health Organization-5 Well-Being Index
WHOQoL-BREF: World Health Organization Quality of Life Assessment
WOMAC: Western Ontario and McMaster Universities Osteoarthritis Index
