# Supplementary material for: Psychometric properties of the EQ-5D-5L: a systematic review of the literature
Source: Qual Life Res. 2020 Dec 7;30(3):647–73. doi: 10.1007/s11136-020-02688-y (PMC7952346; doi:10.1007/s11136-020-02688-y)
Supplement: Supplementary file 1 — Supplementary material 1 (DOCX 65 kb) [file 11136_2020_2688_MOESM1_ESM.docx]

| **Supplementary Table 1: Search and Selection Strategy** | | |
| --- | --- | --- |
| Database | | PubMed, Embase, PsycInfo, EuroQol Webpage |
| Search Criteria | Title/Abstract | EQ-5D 5L, EQ-5D-5L, EQ-5D 5 Level, EuroQol 5L, EuroQol 5 Level |
|  | Language | German AND English |
|  | Publication Date | Original: 2007- May 2016  1^rst^ update: May 2016 - Jan 2018  2^nd^ update: Jan 2018 -Jan 2019 |
| Exclusion criteria | $1  $2  $3  $4  D | no adults or humans  publication language is other than German, English  study does not assess the EQ-5D 5L (if not clear whether 3L or 5L was studied, exclude abstracts published before 2005)  publication type other than primary study, literature review, conference paper  duplicate |

Method

1) Definition of the filter* and keywords / mesh terms

2) Search in PubMed, Embase and PsycInfo

3) Database-based merging of the hits from the individual searches into a working file

4) Merging the hits of the three databases and removing duplicates

5) Search EuroQol webpage

6) Compare the hits of the EuroQol webpage with those in the working file and documentation of hits and duplicates

7) Creation of a literature database with all hits

*Due to the differences in filter functions of the databases used, the number of filters has been reduced to a minimum due to questionable numbers of hits (content-logical check, e.g. the language filter for links with AND and OR). For example, the originally set filters "humans" or "adults" were dispensed with.

**Supplementary Table 2: Characteristics of Included Studies**

| **#** | **First author**  **publication year** | **Sample size** | **Country** | **Study design** | **Sample type** | **% Women** | **Age  mean** ± **SD (range) or % in age groups** |
| --- | --- | --- | --- | --- | --- | --- | --- |
| A1 | Scalone 2011 | 426 | I | Cross-sectional | Chronic hepatitis C, chronic hepatitis B, cirrhosis, liver transplantation and other chronic hepatic diseases | 31.0 | 54.25** ± 16.25 (19-84) |
| A2 | Kim 2012 | T1: 893 (38,5%), T2: 78 (out of 250) | KOR | T1 and 11,5 (6 to 15) days later | Cancer patients aged 18+ yrs receiving chemotherapy at an ambulatory cancer center over a 1-mo period | T1: 50.5  T2: 49.0 | 44.9 ± 15.3 (19-88) 45.3 ± 15.8 (19-88) |
| A3 | Tran 2012 | 1,016 | VT | Cross-sectional survey | Patients with diagnosis of HIV/AIDS based on CD4 count | 36.2 | 35.4 ± 7.0 (NR) |
| A4  A6  A83 | Van Hout 2012*  Janssen 2013  Janssen 2018* | 3,691 | DK, UK, NL, PL, I, SCO (crosswalk) | Cross-sectional | COPD/asthma (n=342), depression (n=250), diabetes (n=284), liver disease (n=645), personality disorders (n=384), rheumatoid arthritis/arthritis (n=372), stroke (n=614), students (n=443) | 52.0 | 51.9 ± 20 (NR) |
| A5 | Augstovski 2013 | 387 | Argentina | Cross-sectional | General population adults living in the Buenos Aries metropolitan area | 48.6 | 39.2 ± 13.7 (NR)  38.9 ± 14.4 (NR) |
| A7 | Keely 2013 | 23 | UK, AU | Qualitative interview study | Professionals with clinical and public health experts, medical doctors and frontline researchers, health economists | 43.5 | NR |
| A8 | Kim 2013 | 600 | KOR | Cross-sectional survey: initial survey and a small re-survey for reliability analysis | Nationally representative general population | 50.5 | 44.9 ± 15.3 (NR) |
| A9 | Lee 2013 | 280 | SG | Test-retest survey: baseline and 1 week after | Patients with histologically confirmed breast cancer | 100.0 | 52.2 ± 9.9 (NR) |
| A10 | Scalone 2013 | 1,088 | I | Cross-sectional | Patients with different chronic heart disorders | 38.0 | NR ± 17.8** (18-89) |
| A11 | Swan 2013 | T1: 109, T2: 71 | USA | Longitudinal study | Patients before and after colonoscopy screening | 53.5 | Women: 62.1  Men: 62.6 |
| A12 | Swinburn 2013 | 100 | UK | Cross-sectional initial assessment survey | Patients with varying levels of psoriasis | NR | NR |
| A13 | Agborsangaya 2014 | 2010: 4,946 (response: 98.7%) 2012: 4,752 (response: 98.8%) | CA | Cross-sectional telephone interview survey | General population | 2010: 52.3; 2012: 55.7 | 46.6 ± 16.5 (18-69) 47.7 ± 17.1 (18-69) |
| A14 | Au 2014 | 105 (34 also completed in-depth interviews) | AU | Cross sectional mixed methods | General public and university affiliated persons | 70.0 | 33.6 ± 12.1 (17-72) |
| A15 | Craig 2014 | 2,614 (91%) completed the EQ-5D instruments | USA | Cross-sectional | Patients with chronic conditions | 49.0 | NR |
| A16 | Garcia-Gordillo 2014 | 133 | ESP | Cross-sectional survey | Patients with parkinson's disease | 28.6 | 64.3 ± 9.74 (34-86) |
| A17 | Hinz 2014 | 2,469 | GER | Cross-sectional | General population | 52.7 | 50.5 ± 17.5 (NR) |
| A18 | Jia 2014 | 645 | CN | Cross-sectional: T1 and 7 days later (T2) | Hepatitis B out- (n=369), and inpatients (n=276) | 25.0 | 43.9 ± NR (18-NR) |
| A19 | Lin 2014 | 670 | USA | Multicenter cross-sectional study | Patients with diagnosis of COPD, 40+ age and with FEV predicted available to indicate disease severity | 42.2 | 68.5 ± 10.4 (NR) |
| A21 | Whitehurst 2014 | 15 | CA | Qualitative focus group study | Individuals with spinal cord injury (from the Vancouver general hospital's spine program) | NR | NR |
| A22 | Alvarado-Bolanos 2015 | 585 | Mexico | Cross-sectional survey | Patients with parkinson's disease | 45.5 | 62.9 ± 12.3 (NR) |
| A23 | Buchholz 2015 | 230 | GER | Longitudinal multicenter study | Inpatient rehabilitation patients: orthopedic (n=114), psychosomatic (n=54), rheumatologic (n=62) | 69.6 | 57.0 ± 12.0 (NR) |
| A24 | Conner-Spady 2015 | 176 | CA | Longitudinal study; 2 mailed questionnaires within a time-span of 2 weeks | Patients with osteoarthritis, who referred to an orthopedic surgeon for total joint replacement | 60.0 | 65.0 ± 11.0 (NR) |
| A25 | Feng 2015 | 5L: 996 | England | Cross-sectional | General population | 5L: 59.3; | < 35: 20.3 %  35–54: 38.3 %  55–64: 15.6 %  >65: 25.9 % |
| A27 | Golicki 2015 | 408 | PL | Cross-sectional | Acute stroke patients during index hospitalization | 48.5 | 69.0 ± 12.9 (23-98) |
| A28 | Golicki 2015 | 112 | PL | Longitudinal: initial hospitalization and 4 mo later | Patients with stroke from 1st week of diagnosis | 51.8 | 70.6 ± 11.0 (39-88) |
| A29 | Greene 2015 | pre=77; post=50 | USA | Longitudinal: pre-operatively and 1-6 yrs after THA | Patients before hip replacement operation (THA), who never had hip arthroplasty | NR | Pre-surgical 63 ± 13  Post-surgical 66 ± 10 |
| A30 | Lou 2015 | 269 | SG | Cross-sectional survey | Breast cancer patients: National Cancer Center and oncology wards of SG General Hospital | 100.0 | 52.1± 9.9 (NR) |
| A32 | Mulhern 2015 | Mobile: 70;  Paper: 66 | Yorkshire UK | Cross-sectional | General adult population (sampled from the longitudinal Yorkshire Health Study) | Mobile: 51.4; Paper:50.0 | Mobile: 44.5 ± 12 (20-64);  Paper 46.2 ± 13.3 (20-65) |
| A33 | O'Leary 2015 | 3,348 | Ireland | Cross-sectional survey | Prostate cancer survivors | 0.0 | <60: 23.9%  60–69: 48.7%  70+: 27.4% |
| A34 | Pan 2014 | 289 | CN | Cross-sectional | Outpatients with type 2 diabetes mellitus (T2DM), not having cognitive impairment | 69.5 | 64.9 ± 9.1 (NR) |
| A35 | Pattanaphesaj 2015 | 117 | TH | Cross-sectional | Outpatient diabetes patients treated with insulin | 62.4 | NR |
| A20  A26  A31  A36  A37  A38  A49  A53  A77  A79  A96 | Mihalopoulos 2014*  Gamst-Klaussen 2015  Mitchell 2015*  Richardson 2015  Richardson 2015  Richardson 2015  Lamu 2016*  Richardson 2016  Engel 2018  Gamst-Klaussen 2018*  Gao 2019* | 8,019 | AU, CA, GER, NOR, UK, USA | Cross-sectional survey | General adults in 6 countries (MIC) | female public: 53.0, female patient: 52.0 | 17.8% 65+ public; 22.6% 65+ patient |
| A39 | Sakthong 2015 | 1,156 | TH | Cross-sectional with re-test | Three hospitals in Bangkok; outpatients aged 17+, who were continuously taking any medicines at least 3 mo to treat their diseases | 48.0 | 50.4 ± 14.4 (NR) |
| A40 | Scalone 2015 | 6,800 | I | Survey with quota sampling | General population | 52.0 | 51.9 ± 17.6 |
| A41 | Shiroiwa 2015 | 1,143 | JP | Cross-sectional | Not representative general population  (sample was younger than GP, because they sampled the same number of people in each age category) | 51.2 | 20–29: 17.3%  30–39: 14.2%  40–49: 16.0%  50–59: 16.6%  60–69: 17.7%  >70: 18.2% |
| A42 | Wang 2015 | 729 | SG | Cross-sectional survey | Persons visiting primary care clinics | English: 46.0; Chinese: 54.8; Malay 64.2 | English 56.31 ± 11.46; Chinese 59.98 ± 10.06; Malay 54.62 ±10.36 |
| A43 | White 2015 | 517 enrolled, 434 patients with valid questionnaires | UK | Multicenter prospective observational cohort: pre-ablation and 8-16 weeks post-ablation | Patients with symptomatic cardiac arrhythmia before and after they have undergone cardiac ablation | 43.0 | 61 ± 12 (NR) |
| A44 | Yang 2015 | 150 | SG | Cross-sectional study | Consecutive patients with a diagnosis of End Stage Renal Disease and on Hemodialysis or Peritoneal dialysis for at least 3 mo | 48.7 | 60.1 ± 11.6 (NR) |
| A45 | Augustovski 2016 | Final inclusion 794 | Uruguay | Cross-sectional | General population, sampling quotas by location | 55.3 | 20-39 48.6%;  40-59: 34.1%;  60+: 17.3% |
| A46 | Chen 2016 | 70 recruited, 65 participated with full data | Taiwan | Longitudinal; Before and after rehabilitation | Stroke patients in rehabilitation | 26.2 | 52.8 ± 11.6 (NR) |
| A47 | Ferreira 2016 | 624 | Portugal | Cross-sectional mixed methods | Students from 2 universities aged 30 yrs or under | 60.4 | 21.7 ± 3.2 (NR-30) |
| A48 | Garcia-Gordillo 2016 | 965 of survey participants | ESP | Cross-sectional survey | Patients with urinary incontinence (self-reported on survey) Spanish National Health Survey 2011-2012 | 30.8 | <18: 0.31%  28-59: 14.09%  60-69: 17.10%  70-79: 29.33%  80+: 39.17% |
| A50 | McCaffrey 2016 | 2,908 | AU | Cross-sectional | General Population (South Australian Health Omnibus Survey (HOS)) | 51.1 | 46.3 ± 18.9 (NR) |
| A51 | Nolan 2016 | 616 baseline, 400 follow-up | UK | Cross-sectional and pre-post study: baseline and 8 weeks after pulmonary rehabilitation | Patients with COPD | 40.3 | 70.4 ± 9.3 (NR) |
| A52 | Oremus 2016 | 48 | CA | Cross-sectional survey | General population in Toronto area | 25.0 | 51.8** ± 24.7** (18-NR) |
| A54 | Rogers 2016 | 92 eligible of 100 respondents | UK | Test-retest survey | Deaf people using British sign language | 69.6 | 18–24: 4.3 %  25–34: 15.2 %  35–44: 18.5 %  45–54: 28.3 %  55–64: 15.2 %  65+: 3.3 % |
| A55 | Wang 2016 | 121 | SG | Cross-sectional | Patients with type II diabetes | 43.0 | 55.5 ± 12.7 |
| A56 | Whitehurst 2016 | 364 | CA | Cross-sectional survey | Adults who have had a spinal cord injury | 37.1 | 50.4 ± 13.2 |
| A57 | Bhadhuri 2017 | 1,587 (36%) | UK | Longitudinal survey: baseline and 12 mo f-up | Family members (carers and non-carers) of Meningitis survivors | 72.0 | 51.1 ± 12.8 |
| A58 | Feng 2017 | JP: 1,026; England: 996; ESP: 1,000 | JP, England, ESP | Cross-sectional survey | Representative general population | JP: 49.8, England: 59.3, ESP: 52.5 | JP: 44.9 ± 14.9; England: 52.1 ± 17.9;  ESP: 43.8 ± 17.3 |
| A59 | Fermont 2017 | 189 | UK | Longitudinal, before and after surgical procedure | Adults with severe and complex obesity before and 6 mo after Bariatric surgery | 75 | 49 ± 10.8** |
| A60 | Huber 2016 | 6,074 | GER | Cross-sectional representative survey | German general population | 52.9 | 47.0 |
| A61 | Konnopka 2017 | 5,007 | GER | Cross-sectional | General population | NR | NR |
| A62 | Nguyen 2017 | 1,571 | VT | Cross-sectional | Randomly selected resident adults of the city of Hanoi | 61.5 | NR (25-44) |
| A63 | Poor 2017 | 238 | HU | Cross-sectional | Psoriasic patients | 37.4 | 47.4 ± 15.2 (18-86) |
| A64 | Yfantopoulos 2017a | 2,279 (22.5%) | GR | Observational survey: data collection in the residence of respondents | Greater Athens area middle-aged and elderly population | 52.1 | 57.3 ± 12.4 |
| A65 | Yfantopoulos 2017b | 396 | GR | part of a multicenter prospective study | Psoriasic patients that were to initiate treatment with calcipotriol plus betamethasone dipropionate in a fixed gel combination under routine clinical practice | 39.9 | 52.0 ± 16.5 |
| A66 | Batt 2018 | 381 | USA | Cross-sectional | Adult male patients undergoing hemophilia treatment who have a history of joint pain or bleeding; Hemophilia treatment centers in the United States (2013-2014) | 0.0 | 35.8** ± 15.5 |
| A67 | Bewick 2018 | 52 | UK | Longitudinal multicenter study: baseline, 12 week f-up, 6 mo f-up | Patients with chronic rhino-sinusitis (clinically confirmed) from UK clinical survey (six centers) | 49.0 | 55.0 (21-81) |
| A68 | Bilbao 2018 | 758 | ESP | Longitudinal: baseline, 6 mo f-up | Patients with hip or knee osteoarthritis from 22 primary care centers in the National Health Service in 3 regions of ESP | 61.9 | 69.8 ± 10.7 |
| A69 | Buckner 2018 | Hemophilia: 299; Caregivers: 150 | USA | Cross-sectional | Adults with hemophilia B, and caregivers of children with hemophilia B | Hemophilia 28.8; Caregivers 77.3 | Hemophilia median 29 |
| A70 | Camacho 2018 | Popoled total 1476 | UK | Cross-sectional with simulated improvement; secondary data analysis | Adults with diabetes, coronary heart disease, severe mental illness, schizophrenia | 44.0 | 48 ± 13.2 |
| A71 | Campbell 2018 | Baseline=21; 9 completed all questionnaires at all measurement time points | AU | Pre-post procedure longitudinal: Before surgery, 3 mo and 1 yr after surgery | Patients undergoing bariatric surgery after long-time waits (many yrs) in Hobart Private Hospital (Tasmania) | 57.0 | 50 ± 10 |
| A72 | Cheung 2018 | 100 | CN | Cross-sectional | Ethnic cantonese speaking Chinese patients, that attended a back pain specialty outpatient clinic | 57.0 | 57 ± 12.5 |
| A73 | Conner-Spady 2018 | 541 Baseline, 537 Follow-up | CA (Manitoba) | Pre-post operation, consecutive patients | Patients with osteoarthritis, who are candidates for primary total joint replacement | 56.0 | 64.1 ± 10.3 |
| A74 | Crick 2018 | PROACTIVE: 495; TeamCare 225 | CA | data from two previously published studies; baseline and 12 mo after | Two patient populations: n=495 adults post-discharge from general internal medicine ward (3L) and n=225 type 2 diabetes patients, who screened positive for depression symptoms (5L) | PROACTIVE: 50.5; TeamCare: 56.0 | PROACTIVE: 62.8 ± 18.6,  TeamCare: 59.8 ± 10.3 |
| A75 | Easten 2018 | 541 | AU |  | Permanent residents of 17 residential aged care facilities across 4 states of AU | 74.5 | 85.5 ± 8.5 |
| A76 | Efthymiadou 2018 | 675 | EU, AU, Brazil, CN, ID, JP, Malaysia, Kyrgyzstan, Philippines, SG, USA | Cross-sectional | Adults with rare conditions (breast cancer, rare cancers, multiple sclerosis, rheumatoid arthritis, rare diseases) | 85.0 | 47 ± 12.8 |
| A79 | Fan 2018 | 1,710 | UK | Prospective: Baseline and 100 days after | Patients with Parkinson’s and Non-Parkinson’s disease, who have android or IOS mobile phones/tablets | Parkinsons: 47.7; Non-Parkinsons: 67.6 | Parkinsons 62.6 ± 7.47 Non-Parkinsons 59.7 ± 7.17 |
| A80 | Ge 2018 | 1,932 | SG | Cross-sectional | General population (Community dwelling adults) | 56.1 | 51.3 ± 17.2 |
| A81 | Hernandez 2018 | 20,587 | ESP | Cross-sectional | General population (Spanish National Health Survey 2011-2012) | 54.3 | 48 ± 18 |
| A82 | Huber 2018 | 3,739 | GER | 3 annual cross-sectional surveys, merged | Population survey (2012, 2013, 2014) | 49.0 | 41.1 ± 15.5 |
| A84 | Kohler 2018 | 224 | India | Longitudinal: Post delivery, 3-7 and 21-30 days post | Women in the postpartum period (directly after to 6 mo after giving birth) in rural areas | 100.0 | 18-20: 45 21-24: 107 25-29: 59 30-36: 13 |
| A85 | Lagendijk 2018 | 764 (68.5) | NL | Cross-sectional Post surgery | Patients, who have undergone breast cancer surgery according to electronic patient files | 100.0 | 51.3** ± 12.6 |
| A86 | Marti-Pastor 2018 | 7,554 | ESP (Catalan) | Continuous cross-sectional study carried out since 2010 | Representative general population | Unweighted: 49.8; weighted: 50.9 | 47.1 ± 18.9 (18-102) |
| A87 | McClure 2018 | 1,927 | CA | Longitudinal: baseline, 1-yr f-up | Adults with type 2 diabetes | 44.9 | 64.7** ± 11.1 |
| A88 | Purba 2018 | 1,056 | ID | Cross-sectional with a re-test for a sub-sample | General representative population of Indonesia | 50.0 | 17-30: 38.4  31-50: 41.8  >50: 19.9 |
| A89 | Szentes 2018 | 229 (85.4%) | GER | Cross-sectional | Outpatients diagnosed with any interstitial lung disease subtypes | 32.7 | 63.2 ± 12.9 |
| A90 | Tamasi 2018 | 109 | HU | Cross-sectional (Consecutive) | Patients with Pemphigus (autoimmune disease) | 64.2 | 57.2 ± 14.8 |
| A91 | Thaweethamcharoen 2018 | 64 | TH | Cross-sectional | Patients on peritoneal dialysis | 31.3 | 63.4 ±16.6 |
| A92 | Whalley 2018 | 40 | UK | Qualitative | Patients with asthma | 62.5 | 37.9 ± 10.7 |
| A93 | Wijnen 2018 | 509 | NL | Clinical trial | Patients with epilepsy in the context of two clinical trials (ZMILE & SMILE) | 53.0 | 16-24: 12.4 25-44: 46.4 45-64: 35.2 ≥65: 5.5 |
| A94 | Chuang 2019 | PE: 1,054; DVT: 1,537 | FR, AT, GER, I, ESP, CH, UK | Prospective clinical study: baseline, 1-mo f-up | Patients with a first-time or recurrent pulmonary embolism (PE) or venous thromboembolism (DVT) | PE: 46.1; DVT: 46.1 | PE: 61.9 ± 17.2 DVT: 59.6 ± 16.7 |
| A95 | Gandhi 2019 | 148 (consecutive sample) | SG | Prospective longitudinal study: pre- and post-surgery | Patients due for cataract surgery | 49.3 | 65.8 ± 8.2  77% >60 yrs |
| A97 | Hernandez 2019 | 279 | UK, FR | Longitudinal cohort (ASTRO-LAB) | Patients with asthma, persevered >6 mo with inhaled corticosteroids and long-acting beta-agonists, younger than 40 yrs | 60.6 | 31.0 |
| A98 | Kouwenberg 2019 | 463; autologous-BR (n=202), implant-based-BR (n=103), without BR (n=158) | NL | Cross-sectional Post surgery | Patients with breast cancer after breast surgery | 100.0 | autologous-BR: 55 ± 9.3,  implant-based-BR: 53 ± 12.2,  without BR: 63 ± 11.9 |
| A99 | Rencz 2019 | 206 | HU | Multicenter cross-sectional survey | Consecutive outpatient patients with Crohn's disease | 45.1 | 34.7 ± 10.5 (18-70) |

AU Australia, AT Austria, CA Canada, CH Switzerland, CN China, DK Denmark, EU Europe, ESP Spain, f-up follow-up, FR France, GER Germany, GR Greece, HU Hungary, I Italy, ID Indonesia, JP Japan, KOR South Korea, mo month, NL The Netherlands, NOR Norway, PL Poland, SCO Scotland, SD standard deviation, SG Singapore, TH Thailand, THR Total Hip Replacement, UK United Kingdom, USA United States of America, VT Vietnam, yr year, yrs years

*Study used a subpopulation of the overall dataset.

**Estimated from reported results (from median, range or interquartile range to mean and standard deviation based on recommendations from Wan et al (2014)[10]).

| **Supplementary Table 3: Pooled Proportion of Endorsements at No Problems in EQ-5D-5L Dimensions** | | | | | |  |  |
| --- | --- | --- | --- | --- | --- | --- | --- |
|  |  |  | **Pooled Proportion** | **(95% Confidence Interval)** | **Quantifying heterogeneity** |  |  |
| **Population Studies n=24** | MO |  | 0.817 | (0.771 – 0.855) | tau^2 = 0.4681; H = 12.68; I^2 = 99.4% |  |  |
|  | SC |  | 0.941 | (0.922 – 0.955) | tau^2 = 0.4734; H = 8.52; I^2 = 98.6% |  |  |
|  | UA |  | 0.826 | (0.780 – 0.865) | tau^2 = 1.0432; H = 6.06; I^2 = 97.3% |  |  |
|  | PD |  | 0.587 | (0.511 – 0.658) | tau^2 = 0.5667; H = 16.86; I^2 = 99.6% |  |  |
|  | AD |  | 0.720 | (0.656 – 0.776) | tau^2 = 0.5380; H = 14.74; I^2 = 99.5% |  |  |
| **Healthy Sample n=3** | MO |  | 0.949 | (0.915 – 0.970) | tau^2 = 0.2016; H = 3.04; I^2 = 89.2% |  |  |
|  | SC |  | 0.993 | (0.971 – 0.999) | tau^2 = 1.0221; H = 2.82; I^2 = 87.4% |  |  |
|  | UA |  | 0.903 | (0.865 – 0.931) | tau^2 = 0.0923; H = 2.87; I^2 = 87.8% |  |  |
|  | PD |  | 0.608 | (0.547 – 0.666) | tau^2 = 0.0443; H = 3.21; I^2 = 90.3% |  |  |
|  | AD |  | 0.590 | (0.454 – 0.713) | tau^2 = 0.2287; H = 6.95; I^2 = 97.9% |  |  |
| **Musculo- skeletal n=11** | MO |  | 0.126 | (0.057 – 0.256) | tau^2 = 2.0206; H = 7.99; I^2 = 98.4% |  |  |
|  | SC |  | 0.506 | (0.380 – 0.631) | tau^2 = 0.7213; H = 6.23; I^2 = 97.4% |  |  |
|  | UA |  | 0.144 | (0.083 – 0.240) | tau^2 = 1.0432; H = 6.06; I^2 = 97.3% |  |  |
|  | PD |  | 0.028 | (0.010 – 0.073) | tau^2 = 2.3065; H = 4.97; I^2 = 96.0% |  |  |
|  | AD |  | 0.439 | (0.391 – 0.487) | tau^2 = 0.0806; H = 2.46; I^2 = 83.4% |  |  |
| **Cancer n=9** | MO |  | 0.641 | (0.562 – 0.713) | tau^2 = 0.2053; H = 3.60; I^2 = 92.3% |  |  |
|  | SC |  | 0.846 | (0.763 – 0.904) | tau^2 = 0.5648; H = 4.45; I^2 = 95.0% |  |  |
|  | UA |  | 0.410 | (0.286 – 0.546) | tau^2 = 0.6521; H = 6.09; I^2 = 97.3% |  |  |
|  | PD |  | 0.286 | (0.195 – 0.398) | tau^2 = 0.5186; H = 5.10; I^2 = 96.1% |  |  |
|  | AD |  | 0.442 | (0.337 – 0.553) | tau^2 = 0.4149; H = 4.97; I^2 = 96.0% |  |  |
| **Diabetes n=5** | MO |  | 0.680 | (0.616 – 0.738) | tau^2 = 0.0784; H = 2.39; I^2 = 82.4% |  |  |
|  | SC |  | 0.905 | (0.840 – 0.945) | tau^2 = 0.3669; H = 3.24; I^2 = 90.5% |  |  |
|  | UA |  | 0.731 | (0.623 – 0.818) | tau^2 = 0.2964; H = 4.08; I^2 = 94.0% |  |  |
|  | PD |  | 0.420 | (0.327 – 0.519) | tau^2 = 0.1861; H = 3.61; I^2 = 92.3% |  |  |
|  | AD |  | 0.648 | (0.556 – 0.730) | tau^2 = 0.1687; H = 3.37; I^2 = 91.2% |  |  |
| **Lung Diseases (n=5)** | MO |  | 0.467 | (0.248 – 0.699) | tau^2 = 1.2113; H = 9.71; I^2 = 98.9% |  |  |
|  | SC |  | 0.823 | (0.580 – 0.940) | tau^2 = 1.8684; H = 10.02; I^2 = 99.0% |  |  |
|  | UA |  | 0.459 | (0.254 – 0.680) | tau^2 = 1.0636; H = 9.37; I^2 = 98.9% |  |  |
|  | PD |  | 0.282 | (0.180 – 0.413) | tau^2 = 0.4027; H = 5.89; I^2 = 97.1% |  |  |
|  | AD |  | 0.498 | (0.422 – 0.575) | tau^2 = 0.1046; H = 3.33; I^2 = 91.0% |  |  |
| **Stroke n=4** | MO |  | 0.284 | (0.151 – 0.469) | tau^2 = 0.5895; H = 3.50; I^2 = 91.8% |  |  |
|  | SC |  | 0.338 | (0.245 – 0.446) | tau^2 = 0.1084; H = 1.86; I^2 = 71.2% |  |  |
|  | UA |  | 0.264 | (0.133 – 0.455) | tau^2 = 0.6586; H = 3.61; I^2 = 92.3% |  |  |
|  | PD |  | 0.292 | (0.117 – 0.563) | tau^2 = 1.2351; H = 4.86; I^2 = 95.8% |  |  |
|  | AD |  | 0.333 | (0.151 – 0.583) | tau^2 = 1.0224; H = 4.46; I^2 = 95.0% |  |  |
| **Mental Health n=4** | MO |  | 0.717 | (0.613 – 0.802) | tau^2 = 0.1994; H = 3.60; I^2 = 92.3% |  |  |
|  | SC |  | 0.899 | (0.801 – 0.952) | tau^2 = 0.5081; H = 4.14; I^2 = 94.2% |  |  |
|  | UA |  | 0.396 | (0.305 – 0.494) | tau^2 = 0.1427; H = 3.34; I^2 = 91.0% |  |  |
|  | PD |  | 0.288 | (0.230 – 0.353) | tau^2 = 0.0700; H = 2.36; I^2 = 82.1% |  |  |
|  | AD |  | 0.102 | (0.064 – 0.160) | tau^2 = 0.2179; H = 2.67; I^2 = 86.0% |  |  |
| **Liver Diseases n=4** | MO |  | 0.721 | (0.703 – 0.738) | tau^2 = 0; H = 1.00; I^2 = 0.0% |  |  |
|  | SC |  | 0.886 | (0.864 – 0.905) | tau^2 = 0.0244; H = 1.57; I^2 = 59.4% |  |  |
|  | UA |  | 0.639 | (0.600 –0.676) | tau^2 = 0.0206; H = 1.97; I^2 = 74.2% |  |  |
|  | PD |  | 0.551 | (0.507 – 0.594) | tau^2 = 0.0254; H = 2.19; I^2 = 79.2% |  |  |
|  | AD |  | 0.509 | (0.459 – 0.559) | tau^2 = 0.0351; H = 2.50; I^2 = 84.1% |  |  |
| **Nervous System n=3** | MO |  | 0.371 | (0.187 – 0.601) | tau^2 = 0.6217; H = 5.80; I^2 = 97.0% |  |  |
|  | SC |  | 0.490 | (0.345 – 0.637) | tau^2 = 0.2197; H = 3.65; I^2 = 92.5% |  |  |
|  | UA |  | 0.214 | (0.125 – 0.341) | tau^2 = 0.2005; H = 2.95; I^2 = 88.5% |  |  |
|  | PD |  | 0.184 | (0.125 – 0.264) | tau^2 = 0.1065; H = 2.06; I^2 = 76.4% |  |  |
|  | AD |  | 0.357 | (0.236 – 0.499) | tau^2 = 0.2178; H = 3.44; I^2 = 91.5% |  |  |
| **Skin Diseases n=3** | MO |  | 0.671 | (0.519 – 0.794) | tau^2 = 0.2935; H = 3.79; I^2 = 93.1% |  |  |
|  | SC |  | 0.852 | (0.793 – 0.897) | tau^2 = 0.0930; H = 1.89; I^2 = 72.1% |  |  |
|  | UA |  | 0.717 | (0.569 – 0.829) | tau^2 = 0.3033; H = 3.71; I^2 = 92.7% |  |  |
|  | PD |  | 0.551 | (0.446 – 0.652) | tau^2 = 0.1174; H = 2.69; I^2 = 86.1% |  |  |
|  | AD |  | 0.456 | (0.255 – 0.672) | tau^2 = 0.6006; H = 5.42; I^2 = 96.6% |  |  |

| **Supplementary Table 4: Pooled Correlation of EQ-5D-5L Dimensions with Other Measures of Health** | | | | |
| --- | --- | --- | --- | --- |
| **Measures** |  | **Pooled Rho** | **(95% Confidence Interval)** | **Quantifying heterogeneity** |
| **Physical/ Functional** | MO | 0.494 | (0.447 – 0.538) | tau^2 = 0.0319; H = 4.07 [3.72; 4.45]; I^2 = 94.0% [92.8%; 95.0%] |
|  | SC | 0.391 | (0.345 – 0.435) | tau^2 = 0.0243; H = 3.59 [3.26; 3.95]; I^2 = 92.2% [90.6%; 93.6%] |
|  | UA | 0.464 | (0.426 – 0.500) | tau^2 = 0.0182; H = 3.15 [2.83; 3.49]; I^2 = 89.9% [87.5%; 91.8%] |
|  | PD | 0.410 | (0.366 – 0.452) | tau^2 = 0.0224; H = 3.45 [3.13; 3.81]; I^2 = 91.6% [89.8%; 93.1%] |
|  | AD | 0.255 | (0.218 – 0.291) | tau^2 = 0.0122; H = 2.64 [2.35; 2.96]; I^2 = 85.7% [81.9%; 88.6%] |
| **Pain/ Discomfort** | MO | 0.419 | (0.363 – 0.472) | tau^2 = 0.0172; H = 2.53 [2.16; 2.98]; I^2 = 84.4% [78.5%; 88.7%] |
|  | SC | 0.319 | (0.248 – 0.385) | tau^2 = 0.0240; H = 2.92 [2.52; 3.39]; I^2 = 88.3% [84.3%; 91.3%] |
|  | UA | 0.494 | (0.440 – 0.545) | tau^2 = 0.0191; H = 2.65 [2.26; 3.10]; I^2 = 85.7% [80.5%; 89.6%] |
|  | PD | 0.636 | (0.586 – 0.681) | tau^2 = 0.0276; H = 3.11 [2.70; 3.59]; I^2 = 89.7% [86.3%; 92.2%] |
|  | AD | 0.379 | (0.315 – 0.441) | tau^2 = 0.0222; H = 2.83 [2.43; 3.29]; I^2 = 87.5% [83.1%; 90.7%] |
| **ADLs** | MO | 0.504 | (0.371 – 0.617) | tau^2 = 0.0508; H = 4.05 [3.37; 4.86]; I^2 = 93.9% [91.2%; 95.8%] |
|  | SC | 0.540 | (0.355 – 0.684) | tau^2 = 0.1065; H = 5.77 [4.98; 6.68]; I^2 = 97.0% [96.0%; 97.8%] |
|  | UA | 0.510 | (0.367 – 0.630) | tau^2 = 0.0682; H = 4.65 [3.94; 5.50]; I^2 = 95.4% [93.5%; 96.7%] |
|  | PD | 0.364 | (0.275 – 0.447) | tau^2 = 0.0154; H = 2.38 [1.86; 3.05]; I^2 = 82.4% [71.1%; 89.3%] |
|  | AD | 0.305 | (0.241 – 0.366) | tau^2 = 0.0057; H = 1.65 [1.22; 2.22]; I^2 = 63.2% [33.1%; 79.8%] |
| **Mental Health** | MO | 0.210 | (0.175 – 0.244) | tau^2 = 0.0034; H = 1.81 [1.52; 2.15]; I^2 = 69.4% [56.7%; 78.3%] |
|  | SC | 0.190 | (0.164 – 0.215) | tau^2 = 0.0010; H = 1.29 [1.05; 1.58]; I^2 = 39.7% [9.4%; 59.9%] |
|  | UA | 0.294 | (0.255 – 0.332) | tau^2 = 0.0054; H = 2.14 [1.83; 2.50]; I^2 = 78.1% [70.0%; 84.1%] |
|  | PD | 0.283 | (0.252 – 0.314) | tau^2 = 0.0027; H = 1.67 [1.40; 2.00]; I^2 = 64.3% [48.9%; 75.1%] |
|  | AD | 0.461 | (0.419 – 0.502) | tau^2 = 0.0121; H = 3.00 [2.64; 3.42]; I^2 = 88.9% [85.6%; 91.4%] |
| **Emotional/ Social** | MO | 0.248 | (0.214 – 0.282) | tau^2 = 0.0094; H = 2.83 [2.50; 3.20]; I^2 = 87.5% [84.0%; 90.2%] |
|  | SC | 0.240 | (0.199 – 0.279) | tau^2 = 0.0137; H = 3.34 [2.99; 3.74]; I^2 = 91.1% [88.8%; 92.8%] |
|  | UA | 0.319 | (0.286 – 0.352) | tau^2 = 0.0093; H = 2.81 [2.48; 3.18]; I^2 = 87.3% [83.8%; 90.1%] |
|  | PD | 0.301 | (0.275 – 0.327) | tau^2 = 0.0049; H = 2.15 [1.86; 2.48]; I^2 = 78.3% [71.1%; 83.7%] |
|  | AD | 0.413 | (0.365 – 0.459) | tau^2 = 0.0264; H = 4.54 [4.14; 4.98]; I^2 = 95.2% [94.2%; 96.0%] |
| **Cognition/ Communication** | MO | 0.190 | (0.130 – 0.247) | tau^2 = 0.0086; H = 2.01 [1.53; 2.63]; I^2 = 75.2% [57.4%; 85.6%] |
|  | SC | 0.203 | (0.133 – 0.271) | tau^2 = 0.0136; H = 2.40 [1.88; 3.08]; I^2 = 82.7% [71.7%; 89.4%] |
|  | UA | 0.232 | (0.151 – 0.311) | tau^2 = 0.0199; H = 2.83 [2.26; 3.55]; I^2 = 87.5% [80.5%; 92.0%] |
|  | PD | 0.218 | (0.169 – 0.265) | tau^2 = 0.0050; H = 1.67 [1.24; 2.24]; I^2 = 63.9% [34.6%; 80.1%] |
|  | AD | 0.262 | (0.191 – 0.331) | tau^2 = 0.0149; H = 2.50 [1.97; 3.18]; I^2 = 84.0% [74.1%; 90.1%] |
| **Vitality/ Fatigue/ Sleep** | MO | 0.304 | (0.217 – 0.386) | tau^2 = 0.0274; H = 4.91 [4.20; 5.73]; I^2 = 95.8% [94.3%; 97.0%] |
|  | SC | 0.258 | (0.161 – 0.351) | tau^2 = 0.0332; H = 5.38 [4.65; 6.23]; I^2 = 96.5% [95.4%; 97.4%] |
|  | UA | 0.381 | (0.291 – 0.464) | tau^2 = 0.0322; H = 5.31 [4.58; 6.15]; I^2 = 96.4% [95.2%; 97.4%] |
|  | PD | 0.369 | (0.317 – 0.418) | tau^2 = 0.0093; H = 2.97 [2.41; 3.66]; I^2 = 88.7% [82.7%; 92.6%] |
|  | AD | 0.351 | (0.308 – 0.393) | tau^2 = 0.0058; H = 2.43 [1.92; 3.08]; I^2 = 83.1% [73.0%; 89.5%] |
| **Clinial/ Biological Measures** | MO | 0.471 | (0.377 – 0.555) | tau^2 = 0.0319; H = 4.07 [3.72; 4.45]; I^2 = 94.0% [92.8%; 95.0%] |
|  | SC | 0.487 | (0.374 – 0.585) | tau^2 = 0.0243; H = 3.59 [3.26; 3.95]; I^2 = 92.2% [90.6%; 93.6%] |
|  | UA | 0.468 | (0.356 – 0.567) | tau^2 = 0.0182; H = 3.15 [2.83; 3.49]; I^2 = 89.9% [87.5%; 91.8%] |
|  | PD | 0.329 | (0.247 – 0.407) | tau^2 = 0.0224; H = 3.45 [3.13; 3.81]; I^2 = 91.6% [89.8%; 93.1%] |
|  | AD | 0.320 | (0.199 – 0.431) | tau^2 = 0.0122; H = 2.64 [2.35; 2.96]; I^2 = 85.7% [81.9%; 88.6%] |
